# Toxic metal(loid) speciation during weathering of iron sulfide mine tailings under semi-arid climate

**DOI:** 10.1016/j.apgeochem.2015.01.005

**Published:** 2015-02-07

**Authors:** Robert A. Root, Sarah M. Hayes, Corin M. Hammond, Raina M. Maier, Jon Chorover

**Affiliations:** Department of Soil, Water and Environmental Science, University of Arizona, Tucson, AZ 85721

**Keywords:** arsenic, lead, zinc, XAS, semi-arid, mine tailing

## Abstract

Toxic metalliferous mine-tailings pose a significant health risk to ecosystems and neighboring communities from wind and water dispersion of particulates containing high concentrations of toxic metal(loid)s (e.g., Pb, As, Zn). Tailings are particularly vulnerable to erosion before vegetative cover can be reestablished, i.e., decades or longer in semi-arid environments without intervention. Metal(loid) speciation, linked directly to bioaccessibility and lability, is controlled by mineral weathering and is a key consideration when assessing human and environmental health risks associated with mine sites. At the semi-arid Iron King Mine and Humboldt Smelter Superfund site in central Arizona, the mineral assemblage of the top 2 m of tailings has been previously characterized. A distinct redox gradient was observed in the top 0.5 m of the tailings and the mineral assemblage indicates progressive transformation of ferrous iron sulfides to ferrihydrite and gypsum, which, in turn weather to form schwertmannite and then jarosite accompanied by a progressive decrease in pH (7.3 to 2.3).

Within the geochemical context of this reaction front, we examined enriched toxic metal(loid)s As, Pb, and Zn with surficial concentrations 41.1, 10.7, 39.3 mM kg^-1^ (3080, 2200, and 2570 mg kg^-1^), respectively. The highest bulk concentrations of As and Zn occur at the redox boundary representing a 1.7 and 4.2 fold enrichment relative to surficial concentrations, respectively, indicating the translocation of toxic elements from the gossan zone to either the underlying redox boundary or the surface crust. Metal speciation was also examined as a function of depth using X-ray absorption spectroscopy (XAS). The deepest sample (180 cm) contains sulfides (e.g., pyrite, arsenopyrite, galena, and sphalerite). Samples from the redox transition zone (25-54 cm) contain a mixture of sulfides, carbonates (siderite, ankerite, cerrusite, and smithsonite) and metal(loid)s sorbed to neoformed secondary Fe phases, principally ferrihydrite. In surface samples (0-35 cm), metal(loid)s are found as sorbed species or incorporated into secondary Fe hydroxysulfate phases, such as schwertmannite and jarosites. Metal-bearing efflorescent salts (e.g., ZnSO_4_·*n*H_2_O) were detected in the surficial sample. Taken together, these data suggest the bioaccessibility and lability of metal(loid)s are altered by mineral weathering, which results in both the downward migration of metal(loid)s to the redox boundary, as well as the precipitation of metal salts at the surface.

## 1. Introduction

Beneficiation of massive sulfide deposits for economic base metals such as Cu, Zn, and Pb results in mine-wastes that can be enriched in unextracted metals and metalloids (Hudson-Edwards et al., 2011; Lottermoser, 2010). Under surficial conditions, mine tailings undergo mineral transformations that alter (and often increase) the bioaccessibility of residual metals (Hayes et al., 2009; Meza-Figueroa et al., 2009). Mine tailings are also characterized by poor soil structure, high soluble salts, high concentrations of phytotoxic elements, and low pH (Hammarstrom et al., 2005; Ye et al., 2002), resulting in a lack of vegetative cover and substantially increased vulnerability to particle dispersion due to wind and water erosion (Mendez and Maier, 2008). As a result, mine tailings from arid and semi-arid climates pose a risk to human health from fugitive dust emissions in to neighboring communities.

Arid and semi-arid conditions diminish the prevalence of perennial surface water and, therefore, the dispersion of acid mine drainage. However, the dry climate also makes it difficult to predict the stability of weathered solid phases based on aqueous thermodynamics from pore water chemistry. The dry conditions are nonetheless well suited for in situ characterization by synchrotron X-ray absorption spectroscopy (XAS). This study examines toxic metal behavior in a legacy mine tailings impoundment at the Iron King Mine and Humboldt Smelter Superfund site (IKMHSS) in central Arizona. The site was added to the National Priorities List in 2008 due to its proximity to the town of Dewey-Humboldt and the elevated (>2000 mg kg^-1^) concentrations of several toxic metal(loid)s, of particular concern were As and Pb (USEPA, 2014).

### 1.1 Weathering Trajectory

Residual sulfide minerals in mine tailings dissolve under oxic surficial conditions and release sulfate, metal(loid)s, and protons. Concurrent dissolution of carbonate and silicate minerals consumes protons, but the relative abundance of these minerals is often too low to prevent progressive tailings acidification (Jambor and Blowes, 1994). These reactions result in pore waters that, depending on pH, can be supersaturated with respect to sulfate salts (e.g. melanterite, PbSO_4_, ZnSO_4_·*n*H_2_O), iron hydroxysulfates (e.g. jarosite, schwertmannite, copiapite), secondary carbonates (e.g. PbCO_3_, ZnCO_3_), and (oxy)hydroxides (e.g. ferrihydrite) (Hudson-Edwards et al., 1996). However few studies have focused specifically on the change in speciation of toxic metal(loid)s that occurs during weathering of mine tailings in arid and semi-arid environments.

A previous study examined the mineral weathering trajectory and speciation of the major redox active elements (Fe and S) in the top 2 m of the IKMHSS tailings (Hayes et al., 2014). The study reported dramatic changes in color (dark gray to orange) and pH (7.3-2.3) with decreasing depth in the top 0.5 m accompanied by changes in texture (fining upward) and mineral assemblage indicating progressive transformation of ferrous iron sulfides to ferrihydrite and gypsum, which in turn weathered to schwertmannite and jarosite. These results were largely consistent with previous studies from more temperate environments (e.g. Alpers and Brimhall, 1988; Bigham and Nordstrom, 2000; Jambor et al., 2000), except for the persistence of ferrihydrite in the near surface acid tailings and the lack of detectable goethite, the thermodynamically predicted weathering product of ferrihydrite. This mineral weathering trajectory directly controls the speciation, lability, and transportability of toxic metal(loid)s found in these tailings.

### 1.2 Speciation and Lability of Toxic Metal(loid)s in Mine Tailings

#### 1.2.1. Selective Sequential Extraction

Despite criticisms (incomplete dissolution of target phase, dissolution of non-target phases, re-precipitation during extractions, etc.), selective sequential extractions (SSE) continue to be comprehensively used to examined geomedia and are readily accessible to researchers allowing an operationally-defined quantitative and qualitative analysis of targeted phases (Bacon and Davidson, 2008; Dold, 2003). Selective extractions are a useful investigative tool to help distinguish surface adsorbed ions, strongly and weakly sorbed ions, and operationally-defined isolated mineral phases within complex heterogeneous natural samples, particularly when coupled with complementary direct observational techniques, such as synchrotron XAS measurements.

#### 1.2.2. Application of XAS

Total toxic metal(loid) exposure is only weakly correlated to the metal(loid) body burden or bioaccessibility (e.g. Casteel et al., 2006; Freeman et al., 1992; McBride et al., 2009). Bioaccessibility and lability of toxic metal(loid)s in mine tailings is controlled by speciation, or local bonding environment, and is influenced by weathering time, mineral assemblage, and climate. The speciation of toxic metal(loid)s in the IKMHSS wastes was probed using XAS, which provides information on the molecular-scale bonding environment (Brown and Sturchio, 2002; Kelly et al., 2008). This study focuses on toxic elements As and Pb, which are harmful to humans even at low concentrations (As: O'Day, 2006; Pb: Canfield et al., 2003) and Zn due to the phytotoxic effects that may stymie remediation efforts (Kopittke et al., 2010).

#### 1.2.3. Arsenic in mine tailings

Arsenic, listed number one on the ATSDR priority list of hazardous substances, is a known toxin and carcinogen even at trace levels, causing a variety of human health problems including diabetes, cardiac and renal disease, immunological suppression, and bronchitis (CDC,2013). Arsenic in mine tailings is generally from weathered arsenic bearing sulfides such as arsenopyrite (FeAsS) (Nordstrom and Alpers, 1999; Nordstrom and Archer, 2003; Smedley and Kinniburgh, 2002). Arsenic speciation and lability in mine tailings has been extensively studied with XAS, particularly in environments associated with acid mine drainage (Corriveau et al., 2011a; Corriveau et al., 2011b; Foster et al., 2003; Hudson-Edwards and Edwards, 2005; Jameson et al., 2006; Kim et al., 2011; Maillot et al., 2013; Savage et al., 2000) and the reader is referred to the recent reviews on the topic (Foster and Kim, 2014). These studies have shown that arsenate is the primary weathering product of FeAsS when oxygen is not limited and important secondary minerals and assemblages include scorodite, amorphous ferric arsenate, bidentate binuclear innersphere sorption complexes on ferrihydrite, and As(V) structurally incorporated (or adsorbed) jarosite.

#### 1.2.4 Lead in mine tailings

Lead is of significant health concern because of the known neurotoxicological impacts, especially pediatric and pre-natal, and is listed number two on the ATSDR priority list of hazardous substances (CDC, 2013). Recently, the CDC lowered the regulatory limit for blood Pb levels from 10 to 5 μg dL^-1^, in response to studies demonstrating a statistical reduction in IQ in children at the higher limit (Canfield et al., 2003). Due to the prevalence of Pb in mine wastes, several studies have employed XAS techniques to study speciation in mine tailings and have illustrated the transformation of PbS to relatively more bioaccessible secondary weathering products including: cerrusite, anglesite, plumbojarosite, and sorbed Pb phases often with Fe or Mn (hydr)oxides (Hayes et al., 2011; Hayes et al., 2012; Meza-Figueroa et al., 2009; O'Day et al., 1998; Ostergren et al., 1999; Schuwirth et al., 2007; Van Damme et al., 2010; Waychunas et al., 2002). Studies of Pb in low pH Fe-rich tailings highlight the importance of plumbojarosite and sorbed species (Hayes et al., 2012; Ostergren et al., 1999). Other studies of semi-arid environments have demonstrated the accumulation of bioaccessible Pb-rich sulfate salts at the surface of mine tailings (Hayes et al., 2012; Meza-Figueroa et al., 2009). Studies of carbonate-rich settings demonstrate the most important secondary phases are carbonate and sorbed species (O'Day et al., 1998; Ostergren et al., 1999).

#### 1.2.5 Zinc in mine tailings

Zinc, which is commonly associated with sulfide ore-derived mine tailings, is generally soluble under acidic conditions, and can be phytotoxic at pore water concentrations exceeding *ca*. 30 μM (2 mg L^-1^), thereby limiting revegetation (Kopittke et al., 2010; Long et al., 2003). The use of XAS has been applied to investigate Zn speciation in mine tailings (Juillot et al., 2006; Juillot et al., 2003; Luxton et al., 2013; Manceau et al., 2004; O'Day et al., 1998; Schuwirth et al., 2007; Van Damme et al., 2010). Principal forms of Zn in ore are sphalerite [ZnS], smithsonite [ZnCO_3_], Zn-bearing oxides [e.g., franklinite (ZnFe)_2_O_4_)], and Zn-bearing silicates [e.g., hemimorphite, Zn_4_Si_2_O_7_(OH)_2_·2H_2_O], which weather to smithsonite or goslarite [ZnSO4·7H_2_O], or Zn-bearing secondary ferric phases of ferrihydrite or jarosite, depending on pH (Jamieson et al., 1999; Vodyanitskii, 2008). Schuwirth et al. (2007) combined SSE and XAS to examine Zn speciation in temperate sulfide mine tailings in the northern Rhineland of Germany, and found Zn-rich phyllosilicates, adsorbed Zn species, and Zn coprecipitated with goethite were evident in both oxidized surface (pH 5.5) and reduced subsurface (pH 7.2) tailings, along with sphalerite in subsurface samples. O'Day et al. (1998) reported the formation of Zn hydroxide and sorbed Zn, depending on total Fe content, but did not find evidence of Zn carbonate phases. Another study examining sediments from a mining impacted watershed found principally ZnCO_3_, tetrahedrally coordinated Zn surface complex, and Zn incorporated in phyllosilicate clays (Van Damme et al., 2010). A study of Zn in arid mine tailings reported the near-complete depletion of ZnS in 50 years in the top 0.5 m and formation of Zn-incorporated phyllosilicate, sorbed species, and ZnSO_4_·nH_2_O in surficial samples (Hayes et al., 2011). Iron(III) (hydr)oxides are high affinity sorbents for Zn in natural systems (O'Day et al., 1998; Panfili et al., 2005; Schuwirth et al., 2007), and XAS data indicate that, when present at low concentration, Zn sorbs preferentially via inner-sphere tetrahedral coordination to ferrihydrite and birnessite (Manceau et al., 2002; Manceau et al., 2004; Toner et al., 2006; Waychunas et al., 2002) in oxic Fe- and Mn-rich environments, respectively.

### 1.3 Offsite transport

Quantifying the amount of metal(loid)s being transported off site from fugitive mine waste dusts, and the vector of metal dispersal, are critical components in assessing risk to humans and proximal ecosystems. Wind is a key mechanism of dispersion in (semi)arid systems, particularly where tailings lack vegetative cover that would act to reduce the ground wind shear vector and resultant particle lofting (Breshears et al., 2003; Csavina et al., 2012). Although metal-laden particulate transport has been demonstrated to decrease logarithmically as a function of distance from the source (Benin et al., 1999), fine particulates (<40 μm) can be transported over regional scales (Csavina et al., 2012; Derry and Chadwick, 2007). Several studies have shown that the concentration of toxins can increase with decreasing effective particle diameter (Beamer et al., 2012; Kim et al., 2011), and in sulfide-bearing mine tailings, up to 80% of toxic metals may be present in the <40 μm size fraction (Romero, et al., 2007). This indicates that the particles with the greatest toxic load may be most vulnerable to wind transport over regional distances (Csavina et al., 2012) and are likely to be inhaled or ingested via the hand to mouth exposure (Plumlee and Morman, 2011; Plumlee and Ziegler, 2007). Additionally, the high rate of evapotranspiration in arid and semi-arid environments can lead to the accumulation of soluble efflorescent salts containing toxic metals at the tailings surface; these small particles are vulnerable to erosive forces and are generally readily bioavailable (Csavina et al., 2012; Hayes et al., 2012; Meza-Figueroa et al., 2009). Secondary sulfate salts, iron (oxy)hydroxides, and jarosites detected in surface tailings tend to be very fine grained (e.g., Desborough et al., 2010), adding to the likelihood that they could be transported by wind and fluvial processes.

In the semi-arid Southwestern US, about half of the annual precipitation occurs during intense summer rains of the North American Monsoon, leading to fluvial dispersion of tailings and episodic dissolution of efflorescent salts with resultant seasonal pulses of meta(loid)s into the environment (Jambor et al., 2000; Kim et al., 2013; Navarro et al., 2008). Such events indicate that surface water is also capable of transporting particulates and dissolved metal(loid)s substantial distances downstream. Although the relative importance of aeolian and fluvial transport are site specific, these are the mechanisms of dispersion for toxic metal(loid)-bearing particles.

### 1.4 Site Description

The Iron King Mine and Humboldt Smelter Superfund site (IKMHSS) is a legacy mine tailings impoundment in central Arizona (Dewey-Humboldt, Arizona, USA) from efforts to exploit a nearby massive sulfide deposit for base metals including Cu, Zn, and Pb and smaller amounts of precious metals. The mining operations resulted in ca. 4 × 10^6^ m^3^ of tailings covering 620,000 m^2^ containing elevated As, Pb, and Zn above regional remediation limits and remains barren and vulnerable to erosion (additional details in Supplementary Material (SM) and Hayes et al., 2014). Tailings were initially hydraulically sluiced into a natural topographic depression without compaction or engineered retention, with dikes later stacked around the perimeter to contain the accumulating tailings. The barren surface tailings are acidic (pH 2-3), contain elevated metal(loid)s, high salt content (electrical conductivity, EC = 6.5–9 dS m^-1^), and are susceptible to wind and water erosion, thus increasing the risk of exposure (Hayes et al., 2014).

A previous study of the IKMHSS established the major mineralogy as a function of depth, where depth was used as a proxy for time in the progression of mineral evolution (Hayes et al., 2014). It was shown that sulfides, dominant in the deepest tailings at circum-neutral pH, weather to form gypsum and ferrihydrite in the top 0.5 m of the tailings. In surficial samples, schwertmannite and jarosite were also detected, accumulating with decreasing pH, along with efflorescent sulfate salts. Surprisingly, ferrihydrite was detected in all samples exhibiting oxidation, even at pH as low as 2.3, pointing to the metastable persistence of this phase outside the predicted environmental conditions favoring its stability in semi-arid environments. In the current study, we present toxic metal(loid) speciation (As, Pb, Zn) within the context of the steep mineral weathering gradient previously described, in an effort to assess the present and future bioaccessibility and translocation of these metal(loid)s, and the associated health risk to the adjacent community.

## 2. Materials and Methods

### 2.1 Sample and Reference Material Collection

The IKMHSS tailings samples were collected by excavating a pit to about 1 m, samples were collected and composited across the pit face for discrete depth intervals on the basis of morphological transitions (color, consistency, etc. details in Hayes et al., 2014). A core extending to 2 m depth was extracted adjacent to the excavated pit to acquire deep tailings and recover the presumed un-oxidized, originally deposited material. Samples were sealed in double bagged low O_2_ diffusion bags and transported on dry ice (-78°C). Samples were sub-sectioned under anaerobic atmosphere (H_2_:N_2_ = 5%:95%) in a vinyl glove box (Coy Laboratory Products, MI) to obtain three representative splits from each depth increment and limit post sampling oxidation.

Splits were analyzed as in Hayes et.al. (2014) for (i) moisture content and particle size; (ii) sieved (<2 mm), lyophilized at -80°C and 130 mbar prior to chemical analysis; (iii) kept field moist, frozen, and in darkness prior to sieving and grinding in preparation for XRD and XAS analysis; or (iv) prepared as thin sections following anaerobic drying at room temperature and vacuum imbedding in metal free epoxy (EPO-TEK 301–2FL; Epoxy Technologies, Inc.). Additional details regarding sample preparation procedures are available in the SM.

Reference materials (for As, Pb, and Zn) were collected from mineral source distributors, whereas others, e.g. Zn and As sorbed jarosite, schwertmannite, and As(III) and As(V) sorbed 6-line ferrihydrite, were synthesized in accordance with published methods and equilibrated with 0.1 mM As, Pb, or Zn for 24 hours centrifuged and washed (Bigham et al., 1990; Cornell and Schwertmann, 2003; Gao et al., 2013; Regenspurg and Peiffer, 2005) (see Table S1 for details). The adsorbed metal(loid) mass (in g kg^-1^), calculated on the basis of loss from solution, was 14 (As^III^, ferrihydrite), 14 (As^v^, ferrihydrite), 9.6 (Pb, hematite) and 3.3 (Zn, ferrihydrite). All reagents used were ACS grade or better. The identities of all references were confirmed by XRD.

### 2.2 Chemical Characterization

Tailings samples were analyzed for bulk chemical and environmental analyses of pH, EC, moisture content, color, texture, and petrographic microscopy as described in Hayes et al. (2014). Total elemental composition was measured by inductively coupled plasma- optical emission spectroscopy (ICP-OES) and inductively coupled plasma- mass spectrometry (ICP-MS) following digestion or fusion. Copper, Zn, As, and Pb were measured by ICP-OES following total digestion (HF, HNO_3_, HClO_4_, HCl), and all other elements were reported from ICP-MS, following fusion with LiBO_2_ and Li_2_B_4_O_7_ (Activation Labs, Ontario CA). Instrumental neutron activation analysis (INAA) was used to confirm As total concentrations. Certified reference materials and quality control samples were digested and analyzed along with the tailings samples with an acceptance range of ± 10% of the certified value to verify precision and accuracy in sample preparation and analysis.

The metal(loid) lability and operationally defined solid-phase speciation were determined using a 6-step SSE procedure modified after Dold (Dold, 2003; Neaman et al., 2004). A composite of the top 25 cm of tailings (material being used in remedial plant growth trials; Solis-Dominguez et al., 2012), as well as samples from the pit and core depth increments (A-G; 0-183 cm) were subjected to the SSE (Table 1; details in SM and Hayes et al., 2014). Briefly, the extractions included: 1) nitrogen sparged deionized water (DI H_2_O), targeting soluble salts e.g. gypsum; 2) 1 M ammonium nitrate (NH_4_NO_3_), targeting easily exchangeable and bioavailable metal(loid)s; 3) 1M ammonium acetate (AAc), targeting non-specifically sorbed ions and acid soluble carbonates; 4) 0.05 M sodium phosphate (NaH_2_PO_4_), targeting specifically adsorbed ligands, e.g. inner sphere As complex on ferric (hydr)oxides; 5) 0.1 M ascorbic acid plus 0.2 M ammonium oxalate (AAO), targeting poorly crystalline Al, Mn, and Fe (hydr)oxides; and 6) citrate-bicarbonate-dithionite (CBD), 0.265 M sodium citrate, 0.1107 M sodium bicarbonate, and 0.1435 M sodium dithionite, targeting reducible crystalline Al, Mn, and Fe (hydr)oxides. The residual was calculated by mass balance from the total elemental analysis described above. The SSE was run in triplicate, with the standard deviation used as the reported error.

### 2.3 Bulk XAS Collection and Data Reduction

Bulk metal speciation was interrogated with synchrotron XAS at the Stanford Synchrotron Radiation Lightsource (SSRL) beam lines 4-1 and 11-2. A double-crystal monochromator (Si [220] crystal, = 90), detuned 40% to reject higher order harmonics was used in conjunction with three 15 cm N_2_ filled ion chambers and a 13-, 32-, or 100-element germanium fluorescence detector. Vertical slits of 2 mm (Zn and As) or 3 mm (Pb) were used for all samples, and horizontal slits were adjusted between 2 and 10 mm to maximize fluorescence signal without saturation of the detector. Measurements for Zn were performed at room temperature, and Pb and As measurements were collected at < 15 K using an Oxford liquid He cryostat operating well below the Debye temperature for Pb (105 K) and As (282 K) (Kittel, 2005) thereby reducing spectral contributions from lattice vibrations (Dalba and Fornasini, 1997; King, 1958) and minimizing beam-induced changes in samples, e.g. oxidation or reduction (Bunker, 2010). A minimum of 3 scans were collected in transmission mode (used for most reference materials) and 3-15 scans were collected in fluorescence mode (used for tailings and sorption samples). Aluminum foil filters were used to minimize the contribution of matrix Fe fluorescence (Pb and As only) and Z-1 filters with soller-slits were used to attenuate elastic and Compton scattering, as appropriate, for fluorescence measurements.

Data reduction was performed by averaging replicate scans in SIXPack (version 1.01) (Webb, 2006) after energy calibration. Energy was calibrated by defining the maximum of the first derivative of metal foil spectra as: As K-edge = 11867 eV, Pb L_III_-edge =13035 eV, and Zn K-edge = 9659 eV. The background signal was subtracted with linear pre-edge parameters: As: -150 eV to -20 eV; Pb: -200 eV to -50 or -30 eV; Zn: -210 eV to -100 eV relative to E_0_) and normalized to the post edge oscillation as described elsewhere (Root et al., 2007). Isolation of As backscattering contributions was accomplished by fitting a cubic spline function to the post-edge extended fine structure absorption envelope, which was then weighted by k^3^ and fit by nonlinear least squares methods in k-space using k = 2 Å^-1^ to k_max_ Å^-1^ in the fit with the OPT program in EXAFSPAK (George and Pickering, 2000; Pickering, 2001; O'Day et al., 2004a; O'Day et al., 2004b; Root et al., 2007 for XAS details). Zinc and Pb post-edge spectra were normalized using a quadratic fit to the EXAFS (150 eV above E0 to the end of EXAFS for bulk spectra and 40 eV to *ca*. 150 eV for μ-XAS) using the SIXPack data analysis package (Webb, 2006). EXAFS oscillations were extracted using cubic spline functions. For both Pb and Zn, a variety of EXAFS extraction procedures were attempted and after iterative fittings, the background subtraction method that consistently gave the best fit statistics, as indicated by reduced χ^2^ in linear combination fits was used for all samples and references for all subsequent analysis. An R-space reduction function (R_bkg_) was applied, which minimizes the contribution from low frequency oscillations (i.e., peaks at low R) associated with physically impossible bond distances (Newville et al., 1993). The final background subtraction parameters were: R_bkg_=1.5 and spline range 1 to 9 or 10 Å^-1^ for Pb and Rbk_g_=1.0 and spline range 1 to 11.5 for Zn, with no spline clamps applied in either case.

### 2.4 XAS Linear Combination Fits

During linear combination fitting (LCF), the total number of references was limited to three or fewer components for each individual fit. The statistical measure, χ^2^, was used to compare the relative “goodness” of successive fits. Previous work has suggested the detection limit of phases in XANES is roughly 5%-10% (Foster et al., 1998; O'Day et al., 2004a), thus components fit with a fraction less than 10% were removed and the spectra were refit to determine if the addition of the low concentration phase significantly improved fit statistics. Best fit determination was accomplished by comparing R-values after fit iterations, and fit sensitivity was tested by comparing fits over larger and smaller spectral regions and as normalized derivative and non-derivative spectra to assure the component was consistently the best fit.

All LCF components were constrained to be non-negative and component sums were not forced to unity. The oxidation state of As was determined with LCFs of XANES by fitting the normalized edge jump using linear least-squares combinations of reference compound spectra with the computer package DATFIT (Pickering, 2001). Fits were performed using arsenopyrite as the reduced sulfide model and ferrihydrite with sorbed arsenite and arsenate as the As(III) and As(V) models. It was recognized that As(V) may be present in multiple phases in the oxic samples, but As XANES is generally inadequate to resolve different As(V)-ferric (hydr)oxide complexes. Fitting of Pb and Zn derivative XANES (Pb: 13020 to 13070 eV; Zn: XANES, 9650 to 10050 eV) and EXAFS (Pb: k-range 3-9 or 10 Å^-1^; *k*^3^-weighting; Zn: *k*-range 2-11 Å^-1^; *k*^3^ weighting) proceeded independently to test for internal consistency in SIXPack.

Lead fits were performed using the cycle fit function in SIXPack (Webb, 2006), initially using a reference library of 12 Pb-bearing phases, previously published (Hayes et al., 2012). Components were added until the fits were statistically improved by less than 10% through the addition of additional spectra (Table S1). Previously described low pH mine tailings sample, T_2.6_, was used as the XAS reference for plumbojarosite due to the high structural disorder of plumbojarosite formed under surficial conditions, leading to low EXAFS amplitude and a high Debye-Waller factor (Hayes et al., 2012). Linear combination fits (LCF) to bulk Zn spectra were initially performed using the entire reference spectral library used in Hayes et al., 2011 (for details on references used in fits see Table S1). Bulk fits were constrained to be non-negative, and not forced to sum to unity. The final fit components were selected after iterative fitting because they consistently resulted in best fits (based on reduced χ^2^).

### 2.5 XAS Shell by Shell Fitting

Theoretical phase-shift and amplitude functions were calculated with the program FEFF (Rehr, 1993) using atomic clusters taken from the crystal structures of arsenopyrite and angelellite, a known As(V) mineral with geometries similar to those expected for absorber backscatterer interactions of As in the tailings. Multiple scattering paths (MS) from As (V)–O (As-O-O-As) tetrahedra were included as they have been shown to improve EXAFS fits beyond the first shell for arsenate compounds (Beaulieu and Savage, 2005; Ona-Nguema et al., 2005). During EXAFS fitting, the values of interatomic distance (R, Å) of the As-O, As-S, and As-Fe shells were allowed to vary. The photoelectron threshold energy shift, ΔE_0_ (eV), was allowed to float as a common parameter during fit iterations, i.e., a single ΔE_0_ parameter was used for all backscatterer paths in a fit. There is a strong correlation between Debye–Waller (σ^2^) and coordination number (N); therefore one of the terms was held constant during EXAFS fitting. One parameter was allowed to adjust in fits, while with the other was assigned a fixed value based on known structures (e.g. N = 4 for As-O in arsenate) or experimental data (σ^2^ from fits to known crystalline compounds, e.g. FeAsS). Based on empirical fits to known As reference compounds, estimated errors were R ± 0.02 Å, N or σ^2^ ± 30% for atoms beyond the first shell (see O'Day et al., 2004b). Samples with As XANES showing measurable arsenopyrite (below 25 cm) were fit with a single variable of linked arsenopyrite paths. The arsenopyrite paths were assigned to adjust as a stoichiometric unit, i.e., not allowed to freely adjust but rather adjusted in response to the molar concentration of arsenopyrite in the sample. This allowed the addition of multiple paths without exceeding the Nyquist criterion of independent fit parameters (N_idp_) or increasing the degrees of freedom

(eq. 1) $$N_{\text{inp}}=2\Delta k\Delta R/\pi$$

### 2.6 X-Ray Fluorescence Mapping

Synchrotron micro-focused X-ray fluorescence (μ-XRF) elemental maps were collected on thin sections at SSRL on Beamline 2-3 using a Si (111) monochromator crystal, a 2-3 μm incident beam cross-section, and a single element vortex detector; where measured fluorescence related to concentrations of excited elements. Images were collected with a pixel step size of 2.5–3.0 μm, a 50 ms dwell time, and an excitation energy of 13000 eV and 13050 eV to isolate the contribution of As K and Pb L_III_ emission. After XRF maps were collected, PCA analysis was applied to the 40k-80k pixel images to locate regions of interest and unique chemical/spectral differences using SMAK (version 1.01, Webb, 2011). Based on the results of this analysis, representative pixel regions were selected for micro-focused XAS analysis using a 2-3 μm beam spot. Spectra were energy calibrated and analyzed as described above for bulk XAS.

### 2.7 Multiple Energy Phase Mapping

Additional mapping analyses were performed at energies across the Fe and As absorption edges in order to map the spatial distribution of specific oxidation states or Fe- and As-bearing phases (details in Mayhew et al., 2011 and Root et al., 2013). Multiple maps were collected for the same spatial region of interest at multiple energies selected to maximize species differentiation (11869, 11872, 11875, and 11880 eV for As; and 7114, 7121, 7124, 7126, and 7137 eV for Fe). These maps can be de-convoluted using PCA analysis and the absorption coefficients of each of the phases identified using micro-XAS to map the spatial distribution of specific elemental species (Fig. S1).

### 2.8 Geochemical Modeling

Equilibrium activity (Pourbaix) diagrams of E_h_ and pH corresponding to theoretical stability fields among solid and aqueous species were computed with the ACT2 program in GWB V7 (Bethke, 1994) using a modified version of the Lawrence Livermore National Laboratory thermodynamic database (thermo.com.v8.r6+; Delany and Lundeen, 1990) augmented with data from Bingham et al. (1996) for schwertmannite, Kashkay et al. (1975) for plumbojarosite, Nordstrom and Archer (2003) for aqueous As species (Table S2). In this geochemical context, Eh was used as an equilibrium modeling proxy for *f*O_2_ in E_h_-pH space for the As, Pb, and Zn systems, with activity: (Fe and S) =10^-3.5^ M, and (As, Pb and Zn) =10^-6^ M at 25°C and 1 atm (details in SM). The coverages of the calculated stability fields were dependent on the model activity inputs of As, Pb, Zn, Fe, S, O_2_, etc. and the range and robustness of thermodynamic solubility constants from the literature. Phases not observed with the spectroscopic techniques employed were suppressed in the model, e.g. orpiment and realgar. While various solubiliti*es* are reported (e.g. ferrihydrite reported = 3.0: Majzlan et al., 2004; and = 5.56 Delanay and Lundeen, 1990), highly cited thermodynamic constants were used when possible to relate the model to similar works. Nonetheless, the activity-activity diagram summarizes the equilibrium calculations with respect mineral solubilities under conditions that represent an environmentally relevant scenario in acid mine tailings.

## 3. Results

### 3.1 Characterization of IKMHSS Tailings

**Figure 1:**
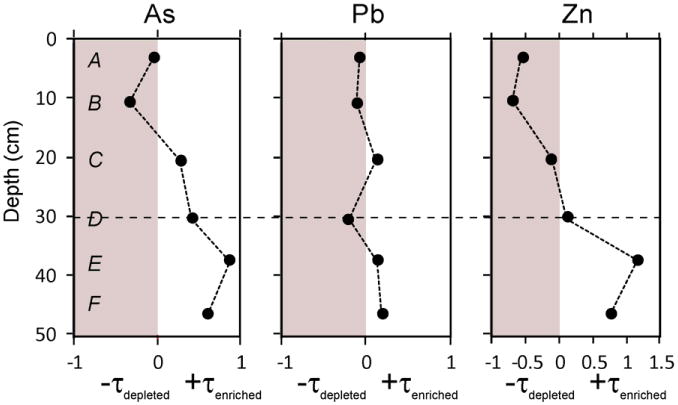
Chemical depletion/enrichment plots showing Ti normalized As, Pb, and Zn mass concentrations in the weathering profile relative to parent material taken as 180 cm sample (see Eq. 2). The horizontal dashed-line represents the redox boundary, whereas the shaded region shows relative depletion.

The IKMHSS near surface tailings exhibited low pH (pH = 2.3), fine grained texture (% sand: % silt: % clay = 15:45:40), elevated salts (EC = 6.5–9 dS m^-1^), and were nearly devoid of neutrophilic heterotrophic bacterial counts (6.7 ± 2.3 × 10^2^ CFU g^-1^) (Hayes et al., 2014; Solis-Dominguez et al., 2012). Whereas the mass concentration of Pb varied only slightly with depth (10.6-13.6 mmol kg^-1^; Table 2), As and Zn concentrations were lower near the surface, increased with depth to a maximum concentration in sample E (35-38 cm at the visible redox boundary), and then decreased again with depth into the “parent material” (represented here by sample G, 180-183 cm). Arsenic and Zn mass concentration in the tailings were strongly correlated with cadmium (As:Cd r^2^ =0.982, p<0.001; Zn:Cd r^2^ =0.982, p<0.01) but not with Pb (As:Pb r^2^ =-0.053 Zn:Pb r^2^ =-0.003). A correlation matrix showing correlation coefficients and p-values for all analyzed elements is given in the SM (Fig. S2). To better constrain enrichment or depletion with depth in the tailings, total elemental concentrations were normalized to Ti, which was assumed to be immobile and redox insensitive across the sampled profile. Figure 1 demonstrates the relative enrichment (+τ) or depletion (-τ) of contaminant metal(loid)s, As, Pb, and Zn as a function of depth relative to the parent material (sample G), according to the following equation (Brimhall and Dietrich, 1987).

[eq. 2] $$\tau_{Ti,j}=\frac{C_{j,w}}{C_{j,p}}\times \frac{C_{Ti,p}}{C_{Ti,w}}-1$$

Where τ_Ti,j_ represents the chemical depletion (if negative) or enrichment (if positive) of element *j* with respect to Ti, *C* represents solid phase mass concentration in the weathering zone (*w*) as measured relative to parent material *(p)*. The τ_Ti_ values for As and Zn show similar trends with moderate depletion in the oxic gossan zone, -0.28 for As and -0.68 for Zn and enrichment below the redox boundary with maximum enrichment of 1.01 and 1.32 for As and Zn, respectively (Fig. 1). Lead was relatively invariant with depth, although a slight depletion was evident in the near surface and at the apparent redox boundary.

**Figure 2:**
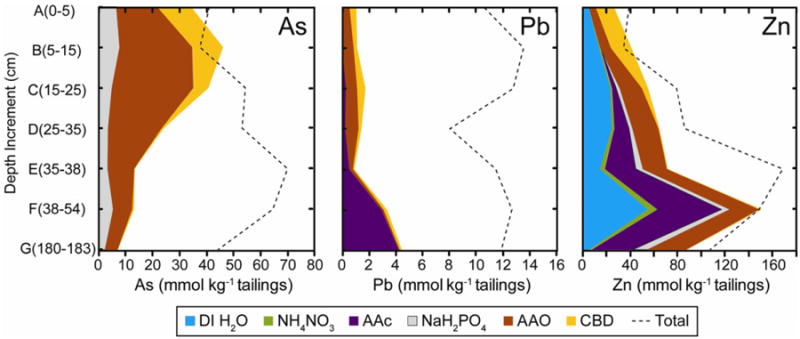
Sequential extraction results for As, Pb, and Zn extractable concentrations in the weathering profile as a function of depth (see online version for color coding). Results in Table S3.

Selective sequential extraction (SSE) results for As, Pb, and Zn are shown in Fig. 2 (full set of numerical results are given in Table S3, including data for Mg, Al, K, Ti, Mn, and Cr). At greater than 35 cm depth, only low levels of As were extracted in the AAc (0.27%-0.64%) and AAO (11%–14%) steps, and most was unextracted or non-labile (79.6%-83.6%). In the upper oxic zone (0-25 cm), SSE extracted As was 75%-123% of the measured total, and was mostly associated with AAO extractable forms (37%-71.8%); 8.7%-19.7% of the As was extracted with NaH_2_PO_4_. The CBD extractable fraction decreased with increasing depth from 30.7% at 0-5 cm to 30.1% at 5-15 cm and 10.1% at 15-25 cm, and <0.2% for all depths below 35 cm.

In all samples, Pb was mostly unextractable (57.9%-91.3% residual) with the 6-step SSE. In the deepest samples (E-G), the largest pool of liberated Pb was from the AAc extraction (23%-35%) which increased with increasing depth. The extractable Pb in A-D (0-25 cm) samples was principally liberated by the AAO (5.1%-12.4%) and CBD (1.9%-4.8%) extraction.

Large pools of Zn were solubilized at all depths by 18MΩ deionized water (5.1%-40.1%) and AAO (12.1%- 30.7%) steps. In the deepest samples (F and G), the AAc extracted 36.3% and 32.3% Zn, respectively. Additionally, extracted Zn from near surface samples (0-25 cm) was from CBD, which decreased with depth from 49.3% at the near surface (5-15 cm) to <1% below 25 cm, with minor Zn liberation from the AAc and NaH_2_PO_4_ extractions in the near surface (0-25cm).

### 3.2 Arsenic Speciation

**Figure 3:**
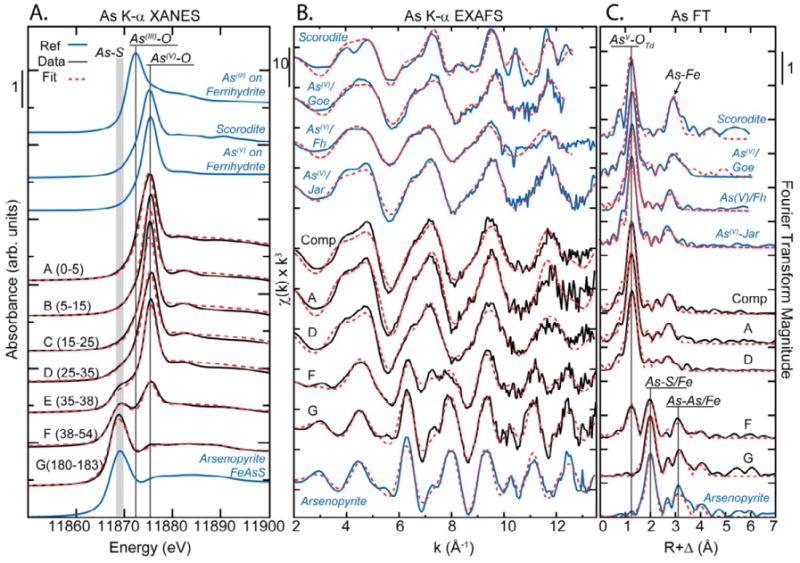
Arsenic K-edge XAS of IKMHSS tailings compared with reference compounds. Solid black lines are data; stippled red lines are least-squares best fits; (A) XANES, (B) unfiltered *k*^3^ weighted EXAFS, and (C) uncorrected for phase shift Fourier transformed EXAFS (FT). Model compounds are shown in blue (see electronic version for color). The vertical band in (A) show reference FeAsS and lines point out As(III)-O and As(V)-O; vertical lines and arrows in (C) highlight the structural features corresponding to the calculated coordination and distance, explained in the text. (A) XANES shows the fit deconvolution with reference arsenopyrite, As(III)–O, and As(V)–O spectra (fit results in Table 3). EXAFS fit results are given in Table 4.

XAS was used to measure the oxidation state and speciation of As as a function of depth. The oxidative weathering transformation from arsenopyrite in deeper tailings to arsenate in surficial samples was quantified using LCFs of XANES spectra (Fig. 3, Table 3). This was possible because of the characteristic edge features at 11869 ±1 eV for sulfides, 11872 ±1 eV for arsenite, and 11875 ±1 eV for arsenate, as previously reported (Foster et al., 1998; O'Day et al., 2004a; Root et al., 2009; Savage et al., 2000). All XANES and EXAFS spectra fits were consistent with mixtures of two components, As(V) sorbed to ferrihydrite and arsenopyrite. The results indicate the presence of only arsenopyrite (102%) by XANES LCF at depth G that is progressively replaced with an increasing mass fraction of As(V) ligated to oxygen (100%-112%) in the near surface (0-25 cm). The XANES fits for the near surface samples of As(V) sorbed to ferrihydrite indicates that the As species in the near-surface tailings was arsenate.

Select depths were analyzed by As EXAFS to investigate the local coordination of the weight averaged As atoms (Fig. 3b, Table 4). At the deepest depth analyzed, 180 cm, the EXAFS spectrum was a close match to arsenopyrite, which has distinctly different spectral characteristics from arsenian pyrite (Fe,As)S_2_ (Savage et al., 2000), which was not observed. EXAFS and XANES analyses agreed that the only As species present in sample G (180-183 cm) was arsenopyrite, which was fit to a model of arsenopyrite with the number of coordinating backscatters (N) fixed allowing the radial distance between backscatters (R, Å) and a common Debye-Waller factor (σ^2^) to vary. The σ^2^ term was thus determined (0.005) and set for arsenopyrite-associated paths in subsequent fits of intermediate depths.

The near surface tailings (sample A) EXAFS spectrum was fit with a first shell of oxygen at 1.68 Å, typical of the As-O_4_ tetrahedra. The second shell was best fit to As-Fe backscatters at 3.29 Å, consistent with bidentate binuclear ^2^C coordination of arsenate to ferrihydrite, a likely coordination environment based on the tailings high Fe content (13 wt%) and the strong affinity of As(V) for ferric (oxy)hydroxides (Dixit and Hering, 2003). The ^2^C coordination has previously been shown to be the dominant mode of As(V) adsorption to octahedra of iron (hydr)oxides including goethite, lepidocrocite, hematite, hydrous ferric oxide (HFO), schwertmannite, and As loaded jarosite (Paktunc and Dutrizac, 2003; Root et al., 2007; Savage et al., 2000; Sherman and Randal, 2003).

Based on XANES fits of sample A (0-5 cm), the structure of As(V) in tetrahedral coordination to four apical oxygen atoms was used to constrain the coordination number (N) in EXAFS analysis by assigning a fixed value of As-O = 4, and allowing σ^2^ to adjust in the fit. For the multiple scattering (MS) paths As-O-O-As within the arsenate tetrahedra, N was likewise fixed at 12 based on path geometry and σ^2^_MS_ was linked to the adjusted σ^2^_As-O_ term for the As-O scattering calculation. The σ^2^ term, determined for As-O in the tailings sample with only arsenate (sample A), was used for subsequent fits for As-O first shell ligands and N was allowed to adjust in the fits.

The As EXAFS spectrum of sample D (25-35 cm) was fit to four oxygen atoms in the first shell at 1.68 Å, but features beyond the first shell were not sufficiently fit with the same interatomic ^2^C distances as the near surface sample. To achieve a good fit to the second shell required two As-Fe distances, 3.28 Å and 3.42 Å, consistent with ^2^C coordination of edge sharing Fe octahedra, and ^2b^C bidentate binuclear ligation to two non-edge sharing Fe (bridging) octahedra, respectively and a contribution from arsenopyrite (∼20%) described above. The σ^2^ term was fixed at 0.005 for all arsenopyrite paths based on the sample G fit.

In sample F (38-54 cm), As was present as mixed species of arsenate and arsenopyrite with the contribution from arsenopyrite more obvious in the EXAFS spectra in the k-range of ∼6-9 Å^-1^. The EXAFS of arsenian pyrite are significantly different from arsenopyrite (Savage et al., 2000) and evidence of arsenian pyrite was not observed. The contribution of arsenopyrite in the As EXAFS was sufficient to explain the spectra from the intermediate depth, however arsenate sorbed to pyrite could not be ruled out. Interestingly, there was no observed As(III) in the tailings samples.

### 3.3 Lead Speciation

**Figure 4:**
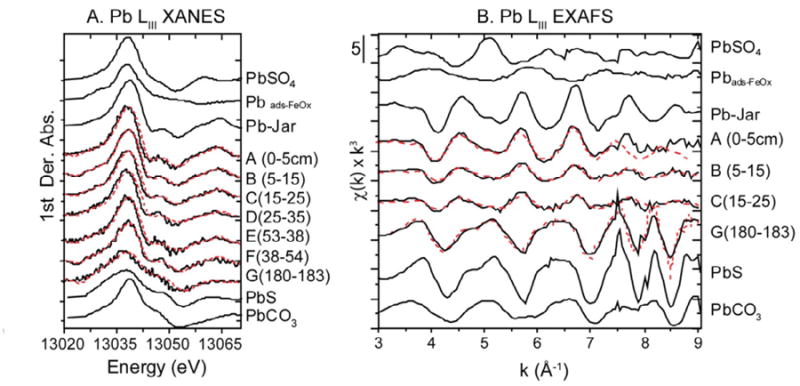
Least-squares linear combination fits of Pb L_III_ in IKMHSS tailings (A) first-derivative XANES, (B) EXAFS. Data are shown by solid black lines and fits are shown by stippled red line. The spectra show that Pb in the tailings weathers from galena to primarily plumbojarosite, but the near surface surficial tailings are enriched in sorbed Pb where pH is >3.

Linear combination fits (LCF) of the derivative XANES and EXAFS revealed Pb speciation changed as a function of depth (Fig. 4, Table 5). Generally, the derivative XANES and EXAFS LCFs were in good agreement, but the sum of the XANES components was closer to unity. XANES fits were performed for all samples, but destructive interference in some EXAFS spectra (samples D-F) resulted in extremely low EXAFS amplitude and precluded collection of quality EXAFS data. Visual examination of the EXAFS reference spectra (Fig. 4) demonstrated that PbS was nearly 180-degrees out of phase with plumbojarosite and other oxidized species, resulting in fits that were non-unique based on comparative reduced *χ*^2^ values.

Galena was the dominant phase in sample G with minor PbCO_3_ (∼20%), accounting for the very broad leading edge feature in the derivative XANES. Above the deepest sample, a strong signal from plumbojarosite was observed at all other depths. Fits of intermediate samples indicated a mixture of PbS, which became depleted with decreasing depth, associated with an increase in plumbojarosite and sorbed Pb associated with Fe (oxy)hydroxides. Samples D-F XANES were fit with plumbojarosite (59%-68%) and galena (40%-21%). Iron (oxy)hydroxide associated Pb, modeled using Pb-sorbed to hematite, was detected in samples B and C (21% and 13%, respectively) in addition to plumbojarosite.

Both EXAFS and XANES indicated that, in surficial tailings (sample A), plumbojarosite was the only XAS-detectable Pb-bearing phase. However, the EXAFS fit total to 121%, indicated that the sample had greater amplitude in the EXAFS region relative to the reference spectrum, which was a plumbojarosite mine tailings sample from a previous study (Hayes et al., 2012). Deviation of the fit totals from unity indicates differences in the degree of order or distribution of bond distances for IKMHSS samples relative to the reference samples employed in the fits. While the XANES LCF indicated plumbojarosite as the sole Pb-bearing phase (103%) the higher mass fraction total EXAFS LCF for sample A indicates increased structural order relative to the reference sample used and plumbojarosite in samples B and C (fit totals 82% and 83%, respectively).

### 3.4 Zinc Speciation

**Figure 5:**
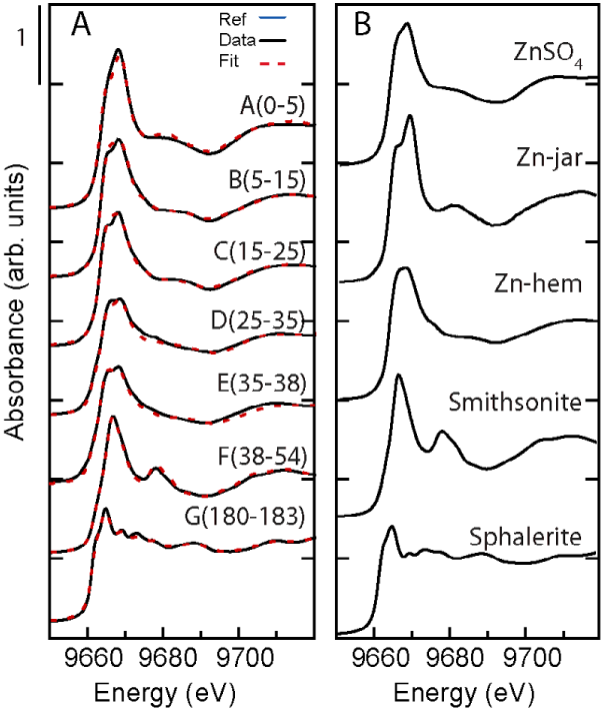
Normalized Zn K-edge XANES collected at 8-15 K for (A) of IKMHSS tailings, and (B) normalized Zn reference compounds: ZnSO_4_=goslarite, Zn-jar =Zn sorbed jarosite, Zn-hem =Zn sorbed hematite, smithsonite = ZnCO_3_, and sphalerite = ZnS (see SM for source and synthesis methods for reference compounds). Data are shown by solid black lines and fits are shown by stippled red line. The XANES fits show that Zn in the tailings weathers from sphalerite to primarily jarosite-adsorbed Zn and goslarite at the surface.

Zinc XANES showed speciation changes due to oxidative weathering as a function of depth in the deep tailings, from ZnS at depth (in parent material) to ZnCO_3_ to Zn sorbed to Fe (hydr)oxides and jarosites, and finally ZnSO_4_·*n*H_2_O in the surface samples (Fig. 5, Table 6). At depth G, Zn was fit to sphalerite (ZnS), but ZnS was depleted to below detection by sample C. Smithsonite was stable under circumneutral to slightly acidic conditions and was found in samples D-F, with the greatest contribution to the XANES fit at depth F (76% to 16%). Smithsonite may have extended to deeper tailings than the increment probed (e.g. 54-180 cm), but was not observed at 180 cm. Iron (oxy)hydroxide associated Zn was detected in samples B-E with a maximum contribution of 94% in sample C, however the specific Fe species (e.g. ferrihydrite, goethite, or hematite) were not distinguishable by Zn XANES alone. Zn adsorption to jarosite minerals was detected in samples A-C (6%-68%) with increasing contribution with decreasing depth. At the surface (sample A), Zn present as a jarosite-adsorbed species (68%) was augmented by its presence in efflorescent salt ZnSO_4_·*n*H_2_O (30%).

### 3.5 Elemental associations with μ-XRF Mapping

**Figure 6:**
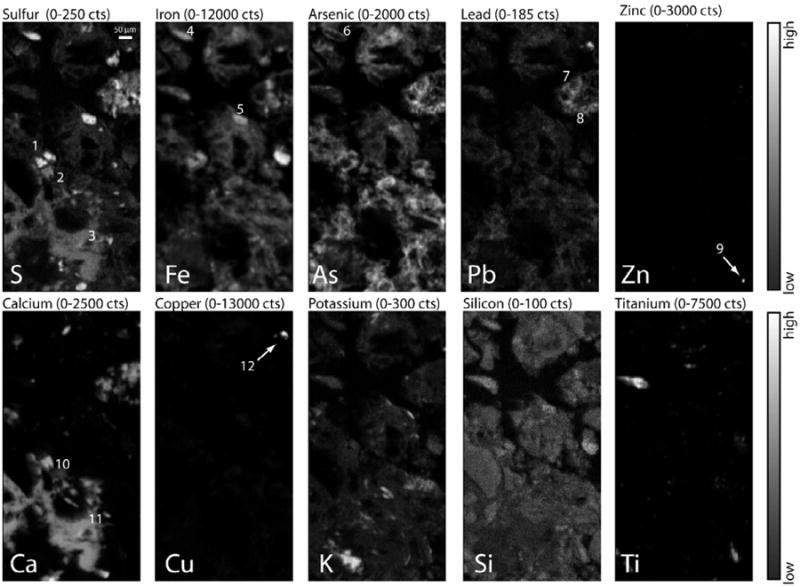
Elemental maps analyzed by μXRF from composited 0-25 cm IKMHSS tailings for S, Fe, As, Pb, Zn, Ca, Cu, K, Si, and Ti. Intensity (black is low, white is high) reported in normalized counts (cts) correlates to concentration with the range (0 – maximum) given at each panel.

To examine the weathering products at the grain-scale and constrain references used in bulk XAS fits, composited (0-25 cm) tailings were examined using μ-XRF mapping (Fig. 6). This technique imaged elemental (S, Fe, As, Pb, Zn, Ca, Cu, K, Si, and Ti) spatial distributions at a 2.5 μm^2^ scale and was used to infer the presence of mineral phases based on co-located elements. The high-Fe high-S tailings (ca. 13 wt% each) led to three distinct Fe-S micro-environments as probed by μ-XRF: (i) sulfidic, characterized by grains of pyrite (Fig. 6, **spots 1, 4, 5**); (ii) acid sulfate, with jarosite (Fig. 6, **spots 2, 7, 8**); and (iii) sulfate salts, dominated by gypsum (Fig. 6, **spot 3**).

**Figure 7:**
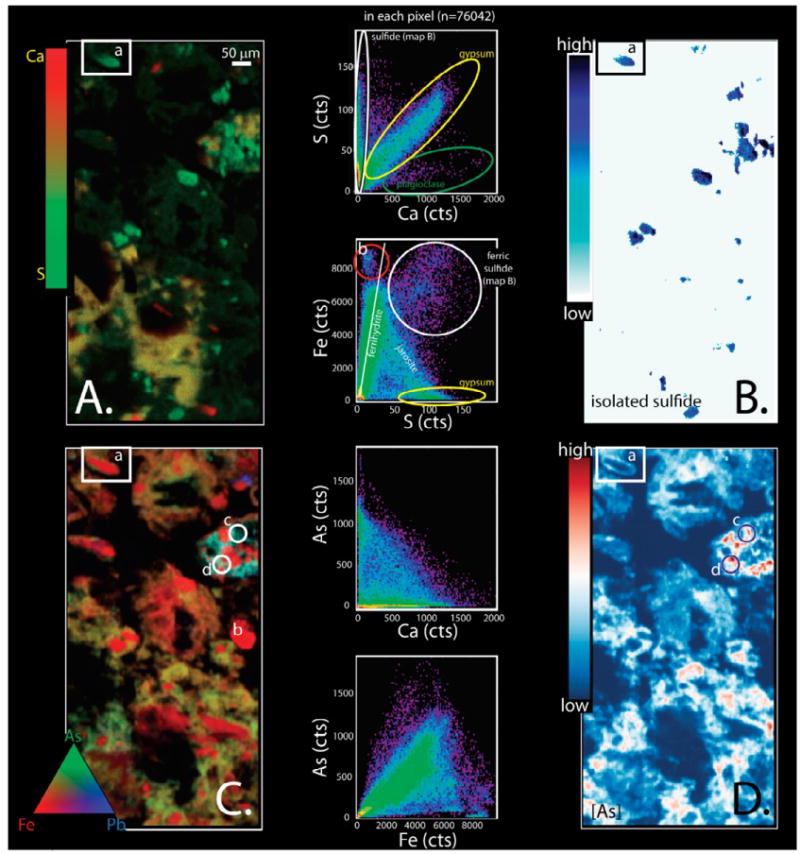
μXRF elemental association maps from 30 μm double polished petrographic thin section from composited 0-25 cm IKMHSS tailings from Fig 6. A.) Ca and S with inset (a) showing grain in Fig. 8. B.) Intensity of isolated sulfide components operationally-defined by masking region of high S and no Ca, encircled in the S v Ca correlation plot. C.) As, Fe and Pb elemental associations, spot (b) refers to the high Fe low S region, (c) and (d) are Pb L_III_ μXANES spots with fits shown in Figure 6 (spots μ-7 and μ-8), fits in and Table 5; D.) relative As concentration (see electronic version for color). Labels on masked regions of correlation plots indicate likely phases based on elemental associations.

**Figure 8:**
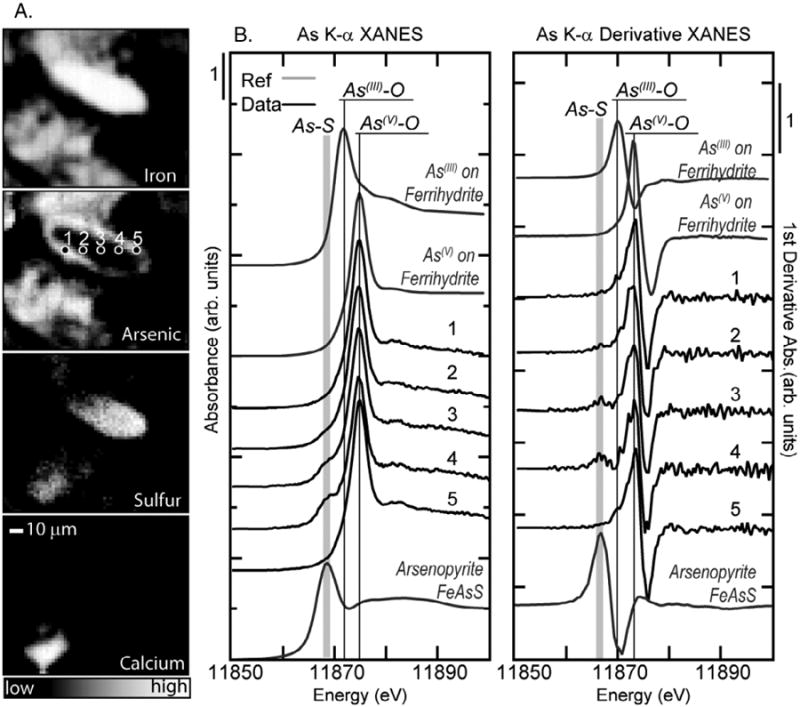
Panels (A) show μXRF elemental intensity images for Fe, As, S and Ca from pyrite grain in Fig. 6 and 7, each map is optimized from minimum to maximum fluorescence counts (black to white); Fe (0-7500 cts), As (0-900 cts), S (0-125 cts), Ca (0-625 cts). Markers 1-5 indicate As μXANES spots across an As(V) rind on a pyrite grain. (B) As K-edge normalized and first derivative μXANES across the pyrite grain. Model compounds are shown in gray lines. The vertical bands show reference FeAsS and lines point out As(III)-O and As(V)-O (gray); fit results in Table 3.

Sulfur and iron are observed throughout the image, but high-concentration bright spots are consistent with pyrite grains (Fig. 6, **spots 1, 4, 5**). Elemental association plots of Fe and S graphically demonstrate distinct regions with relatively high Fe and S intensity (Fig. 7). Arsenic was also observed to be distributed throughout the region sampled, but not as might be expected concentrated in areas with highest co-occurrence of Fe and S counts (e.g. pyrite grains). Rather As was spatially associated with high-Fe areas and formed a rim on the cross-cut pyrite grain in the upper left quadrant (Fig. 6, **spots 4 and 6**; Fig. 8). Close examination of this grain showed a rind of As (Fig. 8), and (μ-XANES at spots (1 through 5) across the grain showed As(V) concentrated on either side of the pyrite grain, and a small contribution of As ligated with S through the interior of the grain (Fig. 8). Lead was concentrated in an area in the upper right quadrant of the image (Fig. 6, **spots 7 and 8, note: same spots as** Fig. 7 **spots μ-c and μ-d**, Table 5), associated with the area mapped as Fe- and S-rich, but not in the pyrite associated areas of highest Fe and S concentrations. The Pb-rich area was also enriched with As.

In contrast to the broader spatial distribution of As and Pb, Zn was observed as a single high-concentration “hot spot” (Fig. 6, **spot 9**). The Zn hot-spot showed no correlation with S, Fe, or As and was assumed to be associated with low-Z elements that are not detected with XRF (e.g. smithsonite). Although fluorescence yield scales non-linearly with atomic number (Z), counts per pixel can be quantitatively compared, keeping in mind the attenuated fluorescence yield at lower Z, and the bright zinc spot with 3000 counts indicated a single grain with a high Zn weight percent or stoichiometry consistent with smithsonite. This was in contrast to the broader spatial distribution of As and Pb.

Calcium distribution showed strong correlation with that of S, specifically in areas that did not also correspond to Fe, consistent with gypsum (Fig. 6, **spots 10 and 11**). Similar to Zn, Cu showed a single “hot-spot”, but in the upper right quadrant. This spot was represented the count intensity maximum and exceeded the maximum intensity of Fe. The co-association of this spot with Fe and S and not As was consistent with the ore mineral chalcopyrite (CuFeS_2_) and not tennatite (Cu_12_As_4_S_13_). Titanium was observed associated with silicon-rich grains, consistent with rutile (TiO_2_) in quartz.

The presence of jarosite minerals, with the generalized formula KFe_3_^III^ (SO_4_)_2_(OH)_6_, was confirmed with XRD and Fe XANES (Hayes et al., 2014). Therefore, XRF should show a per pixel correlation of K with Fe/S if the jarosite present was the K-type. Fluorescence yield increases with atomic number, and a stoichiometric relationship in the intensity counts for analyzed elements relating to an expected mineral is not necessarily expected. However, fluorescence maps can be used to extract elemental ratios, if differences in fluorescence yield for each element is considered. Here, K is only observed in areas of relatively low Fe and S, indicating that S, K, and Fe did not coexist in jarosite at the scale and for the region mapped. This indicates that other jarosite-family minerals (e.g. hydronium [H_3_O^+^], nantro [Na^+^] or plumbo [Pb^2+^]) are likely present in the sample (Bishop and Murad, 2005; Jamieson et al., 2005; Swayze et al., 2000). The distribution of silicon throughout the sample was consistent with the ubiquity of quartz and phyllosilicates, as quantified by XRD (Rietveld refinement, in Hayes et al., 2014)

### 3.6 Arsenic and Iron Speciation with Multiple Energy μ-XRF Mapping

**Figure 9:**
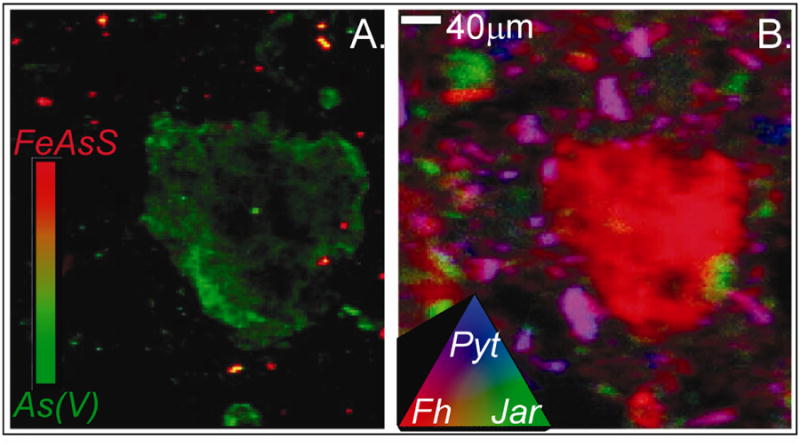
Multiple energy μXRF maps of (A) As species and (B) Fe mineralogy from 30 μm double polished petrographic thin section from sample D.

Fluorescence images compiled from four energies across the As edge and five energies across the Fe edge demonstrate the distribution of corresponding As and Fe speciation at 2.5 μm^2^ pixel resolution for 25-35 cm depth (Fig. 9, sample D). Speciation distribution from the ME μ-XRF maps indicate arsenic was present as two distinct species; As(V) widely distributed and hot-spots of arsenopyrite (Fig. 9A). Iron speciation was dominated by ferrihydrite, with grains of jarosite and pyrite (Fig. 9B). The assigned species were constrained by the fluorescence response in the ME maps across the relevant absorption edge and μ-XANES collected under the same mapping conditions at points of interest determined by PCA (see Root et al., 2013 for details). Comparing the As and Fe maps showed the co-association of As(V) and the ferrihydrite aggregate roughly centered in the images, and the independent pyrite and arsenopyrite grains. The ME μ-XRF maps showed As(V) associated with ferrihydrite and pyrite grains generally separate from arsenopyrite and free of detectible As substitution (i.e., arsenian pyrite).

## 4. Discussion

The fate of toxic metal(loid)s at IKMHSS is controlled by ion mobility and secondary mineral precipitation as a result of primary sulfide and gangue mineral dissolution in the gossan zone. Solid phase products are dominated by ferric iron and sulfate minerals (detailed in Hayes et al., 2014) that, as shown herein, act to buffer the release of toxic elements to the environment. Speciation controls solubility and bioaccessibility of these toxic metal(loid)s, therefore understanding the transformations and translocations in the progressively oxidized weathering zone is key to understanding metal(loid) mobility and risks to surrounding communities and ecosystems (Foster et al., 1998). This study explored the fate of As, Pb, and Zn as a function of depth through the redox zone that occurs within the first two meters of the tailings profile.

Spectroscopic characterization of As, Pb, and Zn by XAS, together with SSE and previously reported quantitative XRD (QXRD by Rietveld analysis), and Fe- and S-XANES (Hayes et al., 2014), showed concomitant changes in speciation along a subsurface redox-driven weathering front that developed from the reaction with atmospheric oxygen. These results showed that the sulfidic component of the deep tailings was composed principally of pyrite, with minor amounts of arsenopyrite, galena, and sphalerite (minor sulfides were <2.5% of total sulfides based on elemental analysis and QXRD) and likely represent the initially deposited waste material.

**Figure 10:**
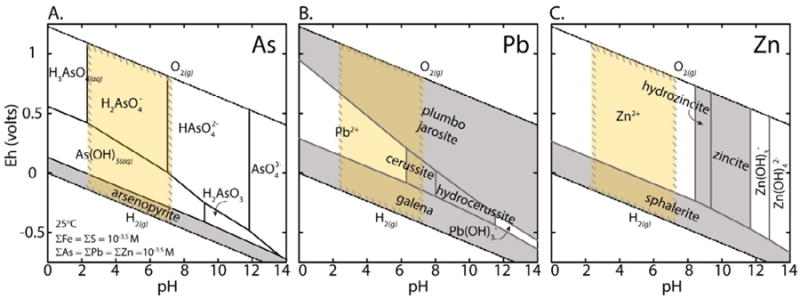
Equilibrium phase relations calculated for total element activities representative of the study site. Eh–pH predominance diagram showing equilibrium among aqueous (un-shaded) and solid phase (shaded) (A) for As; (B) Pb; (C) and Zn at (Fe)_total_ = 10^-3.5^, (S)_total_ = 10^-3.5^, (As)_total_ = 10^-6^, (Pb)_total_ = 10^-6^, (Zn)_total_ = 10^-6^. Phase relations among dissolved species and solid phases calculated for the same system as a function of E_h_ and pH at 25°C and 1 atm, thermodynamic data sources detailed in section 2.8. The hatched area bounds the measured pH at the IKMHSS site and highlights the stability fields. Orpiment and realgar, which were not observed with XAS or XRD, were suppressed in the calculation.

We have characterized the tailings profile (to 2 m) in terms of three distinct zones, the sulfide-rich zone (> 180 cm), a transition (or intermediate) zone that spans the redox gradient (25 – 54 cm), and an oxic (gossan) zone (0-25 cm). The sulfidic parent material likely represent the initially deposited waste material, composed principally of pyrite, with minor amounts of arsenopyrite, galena, and sphalerite (minor sulfides were <2.5% of total sulfides based on elemental analysis and QXRD). The transitional zone (Samples D-F) is defined here as that portion of the profile comprising the primary sulfide form of contaminant metal(loid)s, but having undergone some extent of oxidative transformation, along with the presence of carbonate minerals. Carbonate phases of Pb, Zn, and Fe as (hydro)cerussite, smithsonite, and ankerite were detected in the redox transitional zone but are not detected at pH below 6.3. The onset of oxidative weathering of the initially deposited tailings was evident at 38-54 cm (sample E) below the surface, although it may extend to depths between 54 and 180 cm that were not analyzed. Selective sequential extraction results suggest intermediate depth tailings contain a mixture of sulfides, carbonates, and secondary ferric (oxyhydroxy)sulfates. According to geochemical modeling, these phases are not predicted to co-exist, highlighting the important kinetic (potentially coupled to water through-flux) limitations on oxidative weathering in the semi-arid tailings (Fig. 10). Note that pyrite, the most recalcitrant of the sulfides found in these tailings, is present in all samples, while arsenopyrite, galena, and sphalerite are not detectable in the surficial zone samples (0-25 cm), and it has been shown that toxic metals can be released from sulfide tailings even at low *f*O_2_, and without the concurrent release of acid (Heidel et al., 2013; McKibben et al., 2008). This may be attributed to faster dissolution kinetics of arsenopyrite, galena, and sphalerite relative to pyrite either because of greater specific surface area of the crystallites, or higher chemical affinity. The oxic zone, defined by fine-particle size (45% clay fraction), low pH (< 4) and absence of carbonates, extended from the surface to a depth of 25 cm. Speciation in this portion of the tailings has greatest implications for human health because tailings at the land-air interface are inherently vulnerable to wind shear of fugitive dust and transport into neighboring communities.

### 4.1. Arsenic Weathering

The As XANES and EXAFS indicated only arsenopyrite in the deepest tailings. Importantly, no spectral evidence was found for realgar, orpiment, or arsenian pyrite, which have readily distinguished spectra, even with low-As arsenian pyrite (Foster et al., 1998; O'Day et al., 2004b; Savage et al., 2000). The absence of arsenian pyrite indicates that the sole source As species in the massive sulfide deposit was arsenopyrite that nucleated and grew spatially or temporally separated from the major sulfide pyrite. Arsenopyrite solubility is sensitive to both pH and Eh, and is not predicted to be stable at elevated Eh (Fig. 10).

The highest concentrations of As in these tailings (up to 70.1 mmol kg^-1^) were found in the intermediate transitional zone, indicating that translocation from the relatively depleted surface gives rise to accumulation near the visible redox boundary between orange and gray tailings (Fig. 1). Extractible As in this horizon was limited to 5%-8% with NaH_2_PO_4_ (targeting ligand exchange of inner-spherically adsorbed As), and 11%-37% by AAO (targeting poorly crystalline iron hydroxides hosting occluded As(V)). The XAS results indicate the presence of two distinct As species, FeAsS and iron (oxy)hydroxide-adsorbed As(V) in acidic tailings, represented in the general oxidative dissolution of arsenopyrite (and pyrite) and resultant iron (oxy)hydroxide (as Fe(OH)_3_) precipitation (eq. 3):

(eq. 3) $$2{\text{Fe}}^{\text{II}}{\text{As}}^{0}{S^{-\text{II}}}_{\left(s\right)}+7O_{2}+8H_{2}O\to 2{\left[{\text{Fe}}^{\text{III}}{\left(\text{OH}\right)}_{3}\right]}_{\left(s\right)}+2H_{2}{\text{As}}^{V}{O_{4}}^{-}+2S^{\text{VI}}{O_{4}}^{2-}6H^{+}$$

During the oxidation of arsenopyrite and precipitation of iron (oxy)hydroxide, Fe^2+^ oxidizes to Fe^3+^ (-e^-^), S^2-^ oxidizes to SO_4_^2-^ (-8e^-^) and As^0^ oxidizes to As^5+^ (-5e^-^), with multiple redox species passing through multiple oxidation states that lead to the many varied oxidation products reported in the literature (see Corkhill and Vaughan, 2009).

Analysis of the As EXAFS at 25-35 cm indicate about 20% arsenopyrite, with the remaining As(V) associated with Fe octahedra, apportioned at As-Fe distances of 3.28 Å and 3.42 Å. The distance at 3.28 Å is consistent with a ^2^C bidentate binuclear corner sharing of arsenate tetrahedra with the edge-sharing Fe octahedra. This surface complex is consistent with values reported for As sorbed to ferric (oxy)hydroxide (Table 7, and refs. therein). The longer distance at 3.42 Å indicates As is bridging two non-edge sharing Fe octahedra by corner sharing apical oxygen atoms in a ^2b^C complex, which is close the reported 3.38 Å distance reported for As coprecipitated ferrihydrite by Sherman and Randall, 2003, but not as long as monodentate ^1^V distances (3.57-3.65 Å) reported for goethite and maghemite (Morin et al., 2008; Waychunas et al., 1995). The interatomic differences at the intermediate depth reflect changes in the average local geometry of the sorbent (oxy)hydroxide phase and subsequent bonding of As relative to samples at shallower depths and lower pH. In this study, arsenate was observed associated with iron (oxy)hydroxides (Fig. 9) and absorbed to an iron (oxy)hydroxide rim on a pyrite grain by μ XRF (Fig. 8). Figure 9 demonstrates that As is spatially associated with the ferrihydrite as As(V). However, co-existing, isolated grains of arsenopyrite are also observed. The As(V) is generally not spatially distributed with the arsenopyrite grains, indicating a translocation of As associated with oxidative dissolution.

To examine the variation in As speciation induced by weathering at the grain scale, the raw intensities of the As μXANES from Figure 8 were used to estimate the relative concentrations of different species across a sulfide grain (Fig. 7). The As in the grain interior was 23% - 34% of that in the outer rind (spot 5), indicating a surface enrichment of As(V). The interior of the grain had relatively low total As, mostly As(V), no As(III), and a small shoulder ∼5 eV below the As(V) white line, which indicated that some As was ligated to S (Fig. 8). The As-S ligand accounted for 9% and 17% (fit with arsenopyrite) in spots 3 and 4 (Table 3). The chalcophile surface complex of As on the soft base site of the pyrite grain has been reported (Bostick and Fendorf, 2003), but cannot be differentiated from trace As substitution in the pyrite.

The fate of liberated As from mine tailings has been extensively studied, and surface complexation on pyrite by both As(III) and As(V) (Bostick and Fendorf, 2003; Zouboulis et al., 1993), secondary mineral precipitation [e.g., pitticite (Fe_2_(AsO_4_)(SO_4_)OH·2H_2_O) and pharmacosiderite (2FeAsO_4_·Fe(OH)_3_·5H_2_O); Beattie and Poling, 1987], and the formation of an oxidation layer composed of Fe(III) arsenite and arsenate (Corkhill and Vaughan, 2009) have been reported. Notably absent from any analysis at this site is the presence of arsenite species, suggesting the oxidation of arsenopyrite either (i) progresses as described above (eq. 3), (ii) releases arsenate and arsenite (Walker et al., 2006) but results in preferential re-adsorption of arsenate thereby relatively losing the arsenite to pore water, or (iii) results in a transient As(III) aqueous or solid (colloidal) phase that is rapidly converted to As(V), as reported in laboratory studies (e.g., Asta et al., 2010) and that may not have been captured with the employed methods.

There was near complete release of As during the SSE of the near surface samples (A-C). Most As was removed in the AAO step targeting poorly-ordered secondary ferric phases (37%-72%) and secondarily in the CBD step (10%-31%), whose contribution increased with proximity to the surface, reflecting an increasing proportion of long-range ordered ferric phases with approach to the surface. A slightly smaller NaH_2_PO_4_ extractable pool, reflecting inner-spherically adsorbed As (9%-20%) was also measured in the intermediate samples. XANES spectra of the 0-25 cm composite sample indicate small, but noticeable, changes in the As bonding environment after successive extraction steps, most notably, there was a -0.5 eV change in peak broadening measured as the full width at half the maximum peak height (FWHM) after the AAO extraction removed ferrihydrite (Fig. S3). The sharpening white line peak may be indicative of a lower distribution of atomic arrangements, and would be consistent with for example, As occupation of the tetrahedral site in jarosite. This is further supported by As XANES collected after the CBD extraction, which shows mostly arsenopyrite as a residual species, but a contribution remains from As(V) that is likely due to arsenate sequestered as stoichiometric sulfate substitution in recalcitrant jarosite.

The combination of XAS with extractions indicates that As is principally associated with ferrihydrite-like phase with coprecipitated and/or occluded As with a pool of jarosite associated As that increases with decreasing pH, consistent with findings from similar mine waste sites (Slowey et al., 2007). The ratio of non-stoichiometric As sorbed or occluded with ferrihydrite compared to secondary jarosites is difficult to resolve because the As-Fe distances are very similar and deconvolution of a mixture of both was not possible. When As substitutes for SO4 in the tetrahedral site of jarosite, the As-Fe distance is 3.33 Å (Savage et al., 2000), slightly longer than the 3.29 Å distance expected for ^2^C. The As-Fe ^2^C arrangement was found to be the best fit to the near surface sample (0-5 cm) (Table 7).

Ferrihydrite is a meta-stable phase that is predicted to weather to goethite or jarosite and is thermodynamically unstable at low pH. The long term fate of As adsorbed to ferrihydrite is unknown, and ferrihydrite mineral transformation could potentially release As. Arsenic uptake by jarosite can incorporate ca. 10%-17% of the toxic metalloid (Paktunc and Dutrizac, 2003; Savage et al., 2005), and the maximum loading of As in plumbojarosite is not known. Thermodynamic calculations indicate no predicted As-bearing minerals in the oxidized zone of the mine tailings, but in a semi-arid climate, the meta-stability of high-affinity ferrihydrite surfaces evidently buffer the release of As, maintaining solution phase concentrations below saturation with respect to more soluble and bioaccessible phases like arseniosiderite or ferric arsenate.

The sequestration of As as calcium arsenates (e.g. arseniosiderite, [Ca_2_Fe^III^_3_O_2_(AsO_4_)_3_ • 3H_2_O]) has been reported from tailings in semi-arid regions with abundant gypsum (Foster et al., 2011; Kim et al., 2013). However, the stoichiometric relation of Ca:As in arseniosiderite or yukonite [Ca_2_Fe_3_(AsO_4_)_4_(OH)·14H_2_O] would necessitate a spatial correlation of As and Ca, which was not observed in the 2-3 μm^2^ pixels of the μXRF maps. Corner sharing of As(V) tetrahedra and Ca octahedra has similar second shell features to As-Fe ^2^C in the EXAFS FT, as observed by (Arai, 2010), but was ruled out based on XRD and μ-XRF. Further, EXAFS fits of the IKMHSS samples did not successfully match As-Ca models and the phase was not considered in the final fits. Foster et al. (2011) points out that while there are no thermodynamic data on the stability of calcium arsenates at low pH, the phases may be unstable as conditions become more acidic.

### 4.2 Lead Weathering

The large pool of Pb solubilized by the AAc SSE step (Fig. 2) for the deepest tailings sample is attributed to acid soluble cerrusite (PbCO_3_, log Ksp = -13.13), noted as the most labile form of environmental Pb in swine model bioavailability studies (Casteel et al., 2006). Local atomic structure of the deepest sample probed with Pb L_III_ EXAFS (Fig. 4) shows a very consistent beat pattern with reference mineral galena, but with a slightly dampened amplitude from 3-9 k (Å^-1^) (Fig. 4). The presence of Pb carbonate, which has a similar low-frequency beat pattern in the EXAFS region, but at lower amplitude, accounts for the dampening in sample G relative to pure galena. The first derivative LCF to the Pb XANES correlates with that of the EXAFS, albeit with a lower component sum in the EXAFS. Lead carbonate phases may have been present in oxidized portions of the ore body or may have formed at the time of deposition as the result of exposure to high pH conditions, from the addition of CaO, during flotation (Schlesinger and King, 2011). The lack of carbonate at depths shallower than 180 cm is taken to indicate that PbCO_3_ was present in the original mine wastes and was not diagenetic in the weathering profile. Equilibrium modeling of the Pb system, with (Pb) =10^-6^ M, indicates that at low *f*O_2_ (low Eh) for all ranges of pH (0-14), galena is the predicted stable species (Fig. 10).

At circumneutral pH, and with (HCO3) = 10^-3.5^ M, Pb carbonate is expected to precipitate as cerussite at E_h_ > 0 mV (Fig. 10). Modeling suggests cerussite is metastable with respect to pH, and equilibrium modeling predicts a narrow pH range of stability (6 < pH < 8). This Pb species is, therefore, consistent with the XAS results, geochemical modeling, and the relatively large pool of AAc extractible Pb at the deepest horizon.

Lead was mostly unextracted in the SSE (86%-92%) in intermediate tailings, with the largest pool liberated in the AAO extraction (5%-12%) targeting reducible poorly crystalline Fe, Al, and Mn oxides, indicating the co-association of Pb with iron (oxy)hydroxides. The CBD extraction, targeting reducible crystalline Fe, Al, and Mn oxides, including plumbojarosite, only liberated 2%-5% Pb. Plumbojarosite, the dominant Pb-bearing phase in samples A-F by XAS, has been shown to not dissolve as expected in extractions (Frau et al., 2009; Hayes et al., 2012), partitioning from the solid phases in subsequent extractions (Bacon and Davidson, 2008), or the formation of insoluble Pb (oxalate) phases during extraction, inhibiting Pb release in subsequent steps (Hayes et al., 2009 and refs therein). Thus, the large pool of unextracted Pb in intermediate and surficial samples is likely due to poor plumbojarosite dissolution in the CBD extraction.

In the surficial zone, spectroscopic investigation demonstrates Pb is present exclusively as plumbojarosite (sample A) and mixed with a minor amount of Pb associated with Fe hydroxides in samples B and C (Fig. 4). Plumbojarosite EXAFS spectra have diagnostic features at k= 5 (Å^-1^) and 7.5 (Å^-1^) and the amplitude from k = 4 - 9(Å^-1^) increases with proximity to the surface from C to A due to effective damping due to the low amplitude of the Pb-iron oxide spectra and potentially from an increasingly ordered plumbojarosite. These results, confirmed by (μ-XRF and (μ-XANES, show the presence of Fe, S, and Pb rich grains, consistent with plumbojarosite (Figs. 6 & 7, Table 5). Interestingly, no evidence of bioaccessible anglesite, which has been reported in semi-arid mine tailings, was observed in the SSE or XAS data for the IKHMSS system.

The sequestration of lead in plumbojarosite, a robust phase with sparing bioaccessibility, is an effective mechanism of reducing the environmental risk of Pb exposure. The solubility of plumbojarosite (*log* K_sp_ = -18.3 to -22.84, Chapman et al., 1983; Kashkay et al., 1975) controls Pb transport in the oxic zone of the tailings, and it is several orders of magnitude lower than that of jarosite (*log* K_sp_ = -9 to -12, Bigham et al., 1996; Kashkay et al., 1975). Lead arsenic jarosites (Fig. 7), have a reported solubility (*log* K_sp_ = -13.94) between jarosite and plumbojarosite and this phase is a candidate for long term immobilization of As^5+^ and Pb^2+^ (Forray et al., 2013). The increased stability of plumbojarosite relative to jarosite suggests that the plumbojarosite predominance field would extend to higher pH values. Thermodynamic analysis does not consider kinetic processes; however, the co-existence of ferrihydrite and plumbo-jarosite is possible at equilibrium under conditions found in oxidized mine tailings.

### 4.3 Zinc Weathering

In the deepest tailings investigated (180 cm), 32% of Zn was released in the AAc extraction, indicating the presence of carbonate species, and 31% in the AAO extraction. However, the XANES only indicate the presence of ZnS (Fig. 5) and the spectral fingerprint of Zn reference minerals sphalerite and smithsonite demonstrate unique features in the main edge position (Δ 2eV) and post edge structure (9670-9685 eV) that should allow unambiguous characterization. This may be attributed to transformation of ZnS during the SSE, which was not carried out under anoxic conditions.

Through the transition zone, samples F, E and D showed AAc extractable Zn decreased with proximity to the surface, 36%, 16%, and 14% respectively. This was consistent with the presence of meta-stable smithsonite, which was clearly identified in the Zn XANES, and upward acidification of the tailings. Other Zn-bearing phases identified in the transition zone included ZnS (samples F-D), and Zn-associated with Fe hydroxides (samples D-E). These results are consistent with progressive oxidative weathering in the transition zone.

Specific sequential extractions from the near-surface oxidized samples indicated three Zn-hosting phases, water soluble Zn-sulfate (13%-40%); Zn associated with amorphous iron hydroxide (15%-26%), and Zn extracted with CBD, associated with jarosite (7%-49%). Extractions were consistent with spectroscopic analysis for sample A; Zn was fit to 30% ZnSO_4_·*n*H_2_O, confirming the prevalence of a very soluble, bioaccessible, and phytotoxic species. The observed Zn supergene at and below the redox boundary (4.2 × enrichment over surficial concentrations), combined with the presence of a soluble Zn efflorescent salt at the surface indicate Zn migration both up and down in the tailings profile, likely driven by seasonal hydraulic forces. Soluble salts associated with mine wastes have been reported during dry periods in most climates; however, they are more persistent in arid regions (Bandy, 1938; Hayes et al., 2012; Jambor et al., 2000; Meza-Figueroa et al., 2009). These frequently occur in the form of *A*SO_4_·*x*H_2_O, where *A* is generally a divalent cation such as: Mg, Ca, Fe, Cu and Zn with the general solubility of the divalent hydrated acid sulfates following the trend Ca > Cu > Fe > Mg > Zn.

## 5. Conclusions

Dynamic geochemical conditions in mine wastes develop as sulfide minerals are exposed to atmospheric conditions, with rates of reaction front propagation being and are mainly controlled by *f*O_2_ and hydraulic through flux (percolation downward and capillary rise). Oxidative weathering of sulfide minerals is a function of parent lithology (including the types of sulfides and associated neutralizing minerals), climate, and associated microbial activity. The developed gossan and redox gradient control, to a great extent, the lability of toxic metal(loid)s. This investigation of metal(loid) speciation across the reaction front, combined with a companion investigation of Fe and S speciation (Hayes et al., 2014), serves as a comprehensive mineralogical and geochemical study of weathering of sulfidic mine tailings under semi-arid climate forcing.

Upon exposure to O_2_, oxidative weathering releases As, Pb, and Zn weather from the parent sulfides arsenopyrite, galena, and sphalerite, and incorporates these elements into secondary minerals and sorption complexes of carbonates, (hydr)oxides and (hydroxy)sulfates with a concurrent increase in acidity. By probing speciation with X-ray absorption spectroscopy along the depth-dependent reaction front in the top two meters of tailings, we resolved the transformation of PbS_(s)_ to initially form metastable Fe (oxyhydr)oxide adsorbed Pb species (intermediate in profile), with subsequent incorporation of Pb into stable plumbojarosite in the highly weathered surface tailings. Zinc released from sphalerite weathering was initially incorporated into metastable ZnCO_3_ and Fe (oxyhydr)oxide adsorbed species prior to retention in thermodynamically favored ZnSO_4_ and adsorption complexes with jarosite. Arsenic speciation revealed dominantly a transition from arsenopyrite in initial parent tailings to arsenate adsorbed in inner-sphere complexes with the surfaces of secondary Fe (oxyhydr)oxides and jarosites. Hence, the mobility, and hazardous risk, of toxic elements from tailings impoundments in semi-arid regions is controlled by the speciation of secondary minerals and solid phases involved in surface complexation.

The lability of toxic metals is principally driven via mechanisms of (i) continuous-daily wind and (ii) infrequent-seasonal flash flooding. Predicting toxic exposure is best assessed by understanding speciation and bioaccessibility, which is controlled by the weathering trajectory of the parent mineralogy. Off-site transport is largely influenced by particle size and solubility of secondary minerals, which is a function of speciation and deposition environment controlled by the redox gradient from oxic to sulfidic (surface to depth), pH gradient form very acidic to neutral (surface to depth), and moisture gradient from dry to wet (surface to depth). This report will assist predictions of complex geomicrobiological interaction in managed mine tailings.

## Supplementary Material

supplement

## Figures and Tables

**Table 1:** Selective sequential extraction steps for mine tailings

	Extractant	Conc. (M)	Solid: solution^a^	time temp.	Targeted phase	Reference
1	DI H_2_O N_2_ sparged 18.2 MΩ cm	--	1:30	1 h, 25°C	Soluble salts, e.g. efflorescent sulfate salts	(Dold, 2003)
2	NH_4_NO_3_ Ammonium nitrate pH 7	1.0	1:30	2 h, 25°C	Mobile, easily exchangeable, bioaccessible b	(Merkel, 1996)
3	AAc Ammonium acetate pH 4.5	0.2	1:30	2 h, 25°C	Acid soluble carbonates and non-specifically sorbed	(Dold, 2003)
4	NaH_2_PO_4_ Sodium phosphate pH 5.0	1.0	1:40	24 h, 25°C	Specifically sorbed, e.g. inner-sphere As complexed on ferric (hydr)oxide surface sites	(Keon et al., 2001; Welch and Lico, 1998)
5	AAO Ammonium oxalate pH 3, dark	0.2	1:40	2 h, 25°C	Reducible poorly crystalline Fe, Al, and Mn oxides c	(Dold, 2003; Jackson et al., 1986)
6	CBD Citrate-bicarbonate-dithionite, pH 7, dark	*	1:40	2 h, 80°C	Reducible crystalline Fe, Al, and Mn oxides, e.g. including goethite and jarosite d	(de Koffe et al., 2008; Jackson et al., 1986)

Procedure modified from Dold et al. 2003, results for As, Pb, and Zn in Fig. 2 and tabulated in Table S3.

a mass ratio of solid tailings to extraction solution;

b operationally defined as plant available fraction in Merkel (1996).

c e.g. Schwertmannite and ferrihydrite,

d e.g. secondary jarosite and goethite; the dissolution of plumbo- and other substituted jarosite may not be complete with this sequence (Dold, 2003).

* CBD was 0.265 M sodium citrate, 0.1107 M sodium bicarbonate, and 0.1435 M sodium dithionite at unadjusted pH 8.6.

**Table 2:** Characterization of the Iron King mine tailings.

Sample	Depth^a^ (cm)	pH^b^	As^c^ (mmol kg^-1^)	Pb^c^ (mmol kg^-1^)	Zn^c^ (mmol kg^-1^)	Major^d^ Components	Minor^e^ Components
A	0-5	2.3	41.1	10.6	39.3	qtz, *gyp, jar, fh*	plag, chl, pyt
B	5-15	2.3	37.6	13.6	34.9	qtz, *gyp, fh, jar*	plag, chl, pyt
C	15-25	3.7	54.5	12.8	79.2	qtz, *gyp*, pyt, ill	plag, chl, *fh, schw*
D	25-35	5.5	53.3	8.06	86.3	qtz, *gyp*, pyt	plag, chl, ank, *fh*
E	35-38	6.3	70.1	11.5	168.	qtz, *gyp*, ill, pyt	plag, chl, *fh, sid*, ank
F	38-54	6.0	64.6	12.7	148.	qtz, pyt	plag, chl, ill, *gyp*, *sid*, ank
G	180-183	7.3	44.3	11.9	107.	qtz, pyt	chl, ank, cal

a Depth is the composited interval below the surface;

b pH was measured on wet paste;

c Concentrations of As, Pb, Zn are in mmol kg^-1^ of each in the bulk tailings at each depth sampled.

d Major components are those that make up >10% by XRD Rietveld fits,

e Minor components determined by XRD and XANES.

Phases present in order of estimated abundance (secondary minerals shown in italics): ank=ankerite, cal=calcite, chl=chlorite, *fh=ferrihydrite*, gyp=gypsum, ill=illite, *jar=jarosite*, plag = plagioclase, pyt=pyrite, qtz=quartz, *schw=schwertmannite*.

**Table 3:** Linear combination fit results for As Kα XANES^a^.

Sample	Depth^b^ (cm)	FeAsS^c^ (%)	As^(V)^_ads-Fh_^d^ (%)	c.l.^e^	Σ^f^ (%)
Fig. 3^g^					
A	0-5		100	3.2	100
B	5-15		100	5.0	100
C	15-25		112	4.0	112
D	25-35	9	91	4.1	100
E	35-38	24	84	4.3	108
F	38-54	60	37	1.6	97
G	180-183	102		1.1	102
Fig. 8^h^	0-25				
μ 1			103	1.3	103
μ 2		3	99	2.2	102
μ 3		9	92	2.3	101
μ 4		17	86	2.9	103
μ 5			103	1.5	103

a Results from linear combination least-squares fits;

b composited depth range below surface;

c fit with crystalline arsenopyrite spectrum (O'Day et al., 2004a),

d Fit with arsenate adsorbed to HFO (hydrous ferric oxide precipitated as 6-line ferrihydrite, Gao et al., 2013);

e goodness-of-fit reported as 99% confidence limit (c.l.) defined as three times the estimated standard deviation (Pickering, 2001).

f total of fit component parameters not normalized to unity;

g A-G refers to samples in Fig. 3;

h the shaded region is micro-focused XANES at 2 μm spot size from Fig. 8.

**Table 4:** As K-edge EXAFS fit results^a^

Compound	Atom	*N*	*R* (Å)	*σ*^2^ (Å^2^)	*ΔE_0_* (eV)	*χ*^2^	Sym^a^
*Oxic Zone*							
IK Comp. 0-25 cm	O	4.0^b^	1.68	0.0032	-8.63	4.16	Td
	MS^c^	1.0^b^	3.03/	0.0048/			As-O-O-As
	Fe	1.0	3.29	0.0032/			^2^C
A 0-5 cm	O	4.0^b^	1.68	0.0025	-8.33	4.16	Td
	MS	1.0^b^	3.04/	0.0037/			As-O-O-As
	Fe	1.1	3.29	0.0025/			^2^C
D 25-35 cm	O	4.0^b^	1.68	0.003	-8.00	1.41	Td
	MS	1.0^b^	3.04/	0.0046/			As-O-O-As
	Fe	1.4	3.28	0.003			^2^C
	Fe	1.1	3.42	/			^2b^C/^1^V
	Fe	0.2	2.38	0.005^b^			FeAsS^d^
	As	0.2/	3.07/	/			
	As	0.2/	3.16/	/			
	As	0.4/	3.35/	/			
*Sulfide Zone*							
F 38-54 cm	O	2.24	1.70	0.005^b^	-4.39	1.33	Td
	Fe	1.23	2.38	0.005^b^			FeAsS
	S	0.62/	2.34/	/			
	As	0.62/	3.07/	/			
	As	0.62/	3.17/	/			
	As	1.23/	3.35/	/			
	Fe	1.23	3.78/	/			
G 180-183 cm	Fe	2^b^	2.38	0.005	-5.95	2.30	FeAsS
	S	1^b^	2.34	/			
	As	1^b^	3.07	/			
	As	1^b^	3.17	/			
	As	2^b^	3.35	/			
	Fe	2^b^	3.78	/			
	As	2^b^	*A*4.26	/			

a Results of non-linear least-squares fits; N is the number of backscattering atoms at distance (R); σ^2^, the Debye–Waller term; ΔE_0_ is the threshold energy difference; χ^2^ is a reduced least-squares goodness-of-fit parameter(= F-factor/[# of points - # of variables]). Scale factor (S^2^_0_) = 1.

a Sym is the bulk symmetry or As coordination of absorber-backscatterer pair in sample, explained in text.

b Parameter fixed in least-squares fit using value from fits to reference compounds; / parameter linked in fit to the parameter directly above.

c Spectrum fit with a multiple scattering path from As–O–O–As in arsenate tetrahedra as 12 scattering paths.

d The best fit to the FeAsS structure was achieved by including the first 6 As-N shells, indicated in shaded regions.

**Table 5:** Linear combination fits of derivative Pb L_III_ XANES and EXAFS^a^.

Derivative Pb XANES								
Sample	depth^b^ (cm)	Pb-Jarosite^c^	Pb_ads-FeOx_^d^	PbS^e^	PbCO_3_^f^	red. χ^2^^g^	R-factor^g^	Σ^h^
Fig. 4^i^								
A	0-5	103				0.030	0.017	103
B	5-15	88	13			0.005	0.003	101
C	15-25	81	20			0.016	0.010	101
D	25-35	68		38		0.040	0.029	106
E	35-38	82		21		0.030	0.018	103
F	38-54	59		40		0.022	0.017	99
G	180-183			86	16	0.035	0.033	102
Fig. 7^j^	0-25							
μ7		102				0.033	0.019	102
μ8		98				0.078	0.046	98
Pb EXAFS								
Sample	depth^b^ (cm)	Pb-Jarosite^c^	Pb_ads-FeOx_^d^	PbS^e^	PbCO_3_^f^	red. χ^2^^g^	R-factor^g^	Σ^h^
Fig. 4^i^								
A	0-5	121				0.25	0.13	121
B	5-15	67	15			0.10	0.17	82
C	15-25	61	22			0.16	0.28	83
G	180-183			66	22	0.54	0.12	88

a Results from least-squares fits;

b indicates composited below surface depth range;

c fit with plumbojarosite in mine tailings from a previous study (Hayes et al., 2012,

d Fit with Pb adsorbed to hematite (Hayes et al., 2012);

e fit with crystalline galena;

f fit crystalline with hydrocerussite;

g reduced *χ*^2^ and R-factors are given as goodness-of-fit parameters;

h sum of the fractional fit components not normalized to unity;

i samples A-G refers spectra in Fig. 3;

j the shaded region is micro-focused XANES at spot size 2 μm from spots 7 and 8 from the Pb panel in Fig. 6 and correspond to spots (c) and (d) in Fig. 7.

**Table 6:** Zinc XANES fit results^a^

Sample^b^	depth (cm)	Derivative Zn XANES^a^					Goodness of fit	
	
		ZnSO_4_^c^	Zn-_ads Jar_^d^	Zn-_ads-FeOx_^e^	ZnCO_3_^f^	ZnS^g^	c.l.^h^	Σ^j^
A	0-5	30	68				4	98
*B*	5-15		30	68			<1	98
C	15-25		6	94			3	100
D	25-35			51	16	33	1	100
E	35-38			66	8	30	2	104
F	38-54				76	21	1	98
G	180-183					99	2	99

a Results from least-squares fits.

b sample names and depths are consistent with previous figures and tables;

c Fit with goslarite, representing ZnSO_4_·*n*H_2_O;

d fit with Zn^2+^ adsorbed to synthetic jarosite;

e fit with Zn^2+^ adsorbed to hematite as an iron (oxy)hydroxide surface analog;

f fit with crystalline smithsonite;

g fit with crystalline sphalerite.

h c.l. is the confidence limit, assigned three times the estimated standard deviation for the fit (Pickering, 2001).

i Total of fit components, not normalized to unity.

**Table 7:** EXAFS determined arsenate–ferric hydroxide distances

Mineral	Fe-As (Å)	coordination	note	ref.
Goethite	3.50-3.65	^1^V	m.m.	1, 2
	3.23-3.25	^2^C	b.b.	1, 2
	2.80-2.93	^2^E	m.b.	2, 3, 4
Lepidocrocite	3.29-3.32	^2^C	b.b	1,4,5
Ferrihydrite (HFO)	3.46-3.48	^2b^C	b.b.n.e.	6
	3.16-3.35	^2^C	b.b	1,5,6,7
Schwertmannite	3.27-3.30	^2^C	distorts Schw.	8
Jarosite	3.25-3.35	Td	structural^a^	9, 10,11
Beudantite	1.62 (As-O)^b^	Td	structural	9, 10,11
Scorodite	3.35-3.36	^2b^C	structural	9, 10
IKMHSS tailings^c^	3.29±0.01	^2^C		

References: 1. Waychunas et al., (1995); 2. Fendorf et al., (1997); 3.Manceau (1995); 4. Farquhar et al., (2002); 5. Sherman and Randall (2003); 6. Root et al., (2007); 7. Voegelin et al., (2007); 8. Maillot et al., (2013); 9. Savage et al., (2005); 10. Paktunc and Bruggeman (2010); 11. Majzlan et al., (2014);

a arsenate can reside in the tetrahedral Td site substituting for (SO_4_) in alunite-jarosite group minerals;

b Beudantite has an As-O distance that is much shorter than those observed in the tailings.

c Oxidized mine tailings (top 0-25 cm) from Iron King mine.

^1^V= arsenate tetrahedra corner sharing mononuclear monodentate (m. m.) with apical oxygen from iron hydroxide octahedra, ^2^E = edge sharing mononuclear bidentate (m.b.), ^2^C = corner sharing binuclear bidentate (b.b.) from edge sharing Fe Oh, ^2b^C = corner sharing binuclear bidentate bridging from non-edge sharing Fe (b.b.n.e).

## Highlights

- Large changes in molecular speciation of three toxic metal(loid)s co-occur across a narrow reaction front in the top meter of mine tailings undergoing oxidative weathering.
- Arsenic, released by the oxidation of arsenopyrite, is bound as bidentate binuclear surface complex on ferrihydrite and jarosite in oxidized semi-arid sulfidic mine tailings;
- Lead, released form oxidative weathering of galena, is effectively sequestered as plumbojarosite;
- Zinc weathered from sphalerite is mobilized at pH < 6 as soluble smithsonite and goslarite at pH < 4.
