# Supplementary material for: Toxic metal(loid) speciation during weathering of iron sulfide mine tailings under semi-arid climate
Source: Appl Geochem. Author manuscript; Available in PMC 2016 Nov 1. (PMC4632981; doi:10.1016/j.apgeochem.2015.01.005)
Supplement: supplement [file NIHMS662488-supplement.docx]

**Supplementary Materials**

Toxic metal(loid) speciation during weathering of sulfide mine tailings under semi-arid climate

Robert A. Root^*^, Sarah M. Hayes^†^, Corin Hammond, Raina M. Maier, and Jon Chorover^[[1]](#footnote-1)^

Department of Soil, Water and Environmental Science,

University of Arizona, Tucson, AZ 85721

In preparation for submission to:

*Applied Geochemistry (Sp. Ed for K. Nordstrom)*

* Address correspondence to Robert Root, Department of Soil, Water and Environmental Science, University of Arizona, 1177 E 4^th^ St, Shantz 429, Tucson, AZ 85721

Telephone: +1 520-626-1307, Fax: 520-626-1647, E-mail: robroot.az@gmail.com

Pages: 15

**Table S1:** Sources of Reference Materials

**Table S2:** Thermodynamic Constants for Modeling

**Table S3:** Selective Sequential Extraction Data (As, Pb, Zn, Mg, Al, K, Ti, Mn, Cr)

**Figure S1:** Multiple Energy XANES Mapping

**Figure S2:** Elemental Correlations

**Figure S3:** As difference XANES from SSE

**Sample preparation for XAS and XRF**

Prior to bulk XAS analysis, tailings samples were transported frozen and prepared in an inert atmosphere (H_2_:N_2_ 3:97 Coy glove box). Briefly, 100–200 mg of the field moist sample was hand ground to ca. 5 µm and loaded in a Teflon sample holder, sealed with Kapton tape, and kept under H_2_:N_2_ until data collection.

In preparation for analysis of thin sections by micro-focused mapping, mine tailings were air-dried in the dark in an anaerobic chamber, to minimize photochemical/oxidative reactions, and embedded in metal free epoxy (EPO-TEK 301−2FL; Epoxy Technologies, Inc.). The suspension was cured for 72 h in a vacuum desiccator to purge any occluded air pockets, shipped in a low O_2_ permeability bag (AnaeroGen, Hampshire, England), and thin-sectioned (30 μm, polished 2-sides) under anoxic conditions (Spectrum Petrographic, WA). Thin sections were transported

**Mining site history**

The Iron King Mine and Humboldt Smelter Superfund site (IKMHSS) is a legacy mine tailings impoundment in central Arizona. The tailings pile was constructed using perimeter dike stacking, where hydraulically sluiced tailings from the mine milling facility were pumped and spread, initially in a natural depression, without compaction or engineered retention, thereby building up the pile by subsequent accumulation. The As, Pb, and Zn in the IKMHSS tailings were from arsenopyrite (FeAsS), galena (PbS), and sphalerite (ZnS) from a mined massive sulfide deposit discovered in 1880 ([Creasey, 1952](#_ENREF_3)). The ore was a massive sulphide replacement of schist along defined en echelon veins. Part of a mineral belt cross cutting the Bradshaw Mountains in Central Arizona, the regionally prospected ore was characterized by replacement deposits in Precambrian host rock, rich in gold, silver, and base metals. The Iron King (IK) deposit, within this mineral belt, exploited a massive sulfide deposit, producing principally lead and zinc, with subordinate copper, gold, and silver (details in Hayes et al., 2014). The IK deposit was a system of 12 massive sulfide veins arranged en echelon in a mylonitized shear zone in strongly foliated andesitic-basaltic tuffs (Precambrian Yavapai Series)([Anderson and Creasey, 1958](#_ENREF_1)). The meta-andesite tuffs were fine- to medium-grained chlorite, saussuritized feldspar, and quartz, with the finer-grained facies mostly composed of chlorite. Ore veins consisted of fine-grained, massive sulfides held together by a gangue of ankerite, quartz, sericite, and residual chlorite. The veins were 1 to 14 feet in width and ranged from 150 to 500 feet long; the entire mineralized zone was 75 feet wide. Two groups of veins were recognized in the deposit; all but a few tons of ore came from the well-defined massive sulfide veins, additional mining came from poorly defined veins, chiefly of pyrite, ankerite, and quartz. The massive sulfide veins comprise thin layers of fine-grained pyrite, sphalerite, chalcopyrite, arsenopyrite, galena, and tennatite. Quartz and ankerite were the major gangue minerals; gold was associated with the pyrite and silver was probably in the tennatite ([Koschmann and Bergendahl, 1968a](#_ENREF_10); [Koschmann and Bergendahl, 1968b](#_ENREF_11))). The host rock within the veins was nearly completely replaced and the sulfides had sharp wall rock contacts. Post processing residual sulfides, left as waste mine tailings dissolved under oxic surficial conditions and released sulfate, metal(loid)s, and protons. The barren surface tailings are acidic (pH 2-3), have elevated metal(loid)s, high salt content (EC 6.5–9 ds m^-1^), and are susceptible to wind and water erosion; thus increasing the risk of environmental and health problems in neighboring communities and ecosystems. The site was added to the National Priorities List in 2008 due to its proximity to the town of Dewey-Humboldt and the elevated (>2000 mg kg^-1^) concentrations of several toxic metal(loid)s, of particular concern were As and Pb ([USEPA, 2014](#_ENREF_15)).

**Table S1**: Sources of Reference Materials.

| **Mineral** | **Formula** | **Source** | **Lot/locality** | **Spectra Reference** |
| --- | --- | --- | --- | --- |
| angelellite | Fe^III^_4_(AsO_4_)_2_O_3_ | Synthetic; Berdesinski, 1960 |  | unpublished |
| arsenopyrite | FeAsS | R. Root collection | Ontario, Canada | [O'Day et al., 2004](#_ENREF_14) |
| scorodite | Fe^II^AsO_4_ •2H_2_O | UCM collection | Goldhill, Utah | [O'Day et al., 2004](#_ENREF_14) |
| anglesite | PbSO_4_ | Aldrich | 0731BE | [Hayes et al., 2012](#_ENREF_6) |
| cerrusite | PbCO3 | Wards | 1360 | [Hayes et al., 2012](#_ENREF_6) |
| galena | PbS | Sigma Aldrich | 00508MH | [Hayes et al., 2012](#_ENREF_6) |
| plumbojarosite | Pb(Fe_3_(SO_4_)_2_(OH)_6_)_2_ | UA Mineral Museum | 5751 | [Hayes et al., 2012](#_ENREF_6); [Hayes et al., 2009](#_ENREF_7) |
| goslarite | ZnSO_4_∙ 7H_2_O | Mallinckrodt | V07616 | [Hayes et al., 2011](#_ENREF_5) |
| smithsonite | ZnCO_3_ | Wards | 5965 | [Hayes et al., 2011](#_ENREF_5) |
| sphalerite | ZnS | Wards Scientific | Picos de Europa, Spain | [Hayes et al., 2011](#_ENREF_5) |
| chlorite | (Mg, Fe)_5_Al(Si_3_Al)O_10_(OH)_8_ | Clay Minerals Repository | CCa-2 | [O'Day et al., 2004](#_ENREF_14) |
| ferrihydrite | Fe_2_O_3_·2FeOOH·2.6H_2_O | Synthetic; Burleson and Penn (2006) |  | Gao et al., 2013 |
| hematite | α-Fe_2_O_3_ | Synthetic; Schwertmann and Cornell 1991 |  | [O'Day et al., 2004](#_ENREF_14) |
| jarosite | KFe^III^_3_(SO_4_)_2_(OH)_6_ | Synthetic; Driscoll et al., 2005 |  | Hayes et al., 2014 |
| pyrite | FeS_2_ | Wards Scientific |  | [O'Day et al., 2004](#_ENREF_14) |

**Table S2:** Thermodynamic data for conditions in geochemical Eh-pH diagrams

log *K_sp_*

*Iron solid*

Jarosite [Fe_3_(SO_4_)_2_(OH)_6_]+ 6H^+^ 🡘 3Fe^3+^ + 2SO_4_^2-^ + K^+^ + 6H_2_O -9.37^a^

Schwertmannite

[Fe_8_O_8_(OH)_4.8_(SO_4_)_1.6_] + 20.8H^+^ 🡘 8Fe^3+^ + 12.8H_2_O + 1.6SO_4_^2-^ -17.4^b^

Ferrihydrite [Fe(OH)_3_]+ 3H^+^ 🡘 Fe^3+^ + H_2_O 3.0^c^

[Fe(OH)_3_] + 3H^+^ 🡘 Fe^3+^ + H_2_O 5.56^a^

Pyrite [FeS_2_]+ H_2_O 🡘 Fe^2+^ + 0.25SO_4_^2-^ + 1.75HS^-^ + 0.25H^+^ 24.65^a^

*Zinc solid*

Smithsonite [ZnCO_3_] + H^+^ 🡘 HCO_3_^-^ + Zn^2+^ 0.46^d^

Sphalerite [ZnS] + H^+^ 🡘 Zn^2+^ + HS^-^ 11.44^d^

Goslarite [ZnSO4·7H_2_O] 🡘 Zn^2+^ + SO_4_^2-^ + 7H_2_O 1.87^e^

*Lead solid*

Anglesite [PbS] 🡘Pb^2+^ + SO_4_^2-^ 7.85^d^

Plumbojarosite 22.84^f^

[PbFe_6_(SO_4_)_4_(OH)_12_ + H^+^ 🡘 Pb^2+^ + SO_4_^2-^ + Fe^2+^ + 12H_2_O

Cerrusite [PbCO_3_] + H^+^ 🡘 HCO_3_^-^ + Pb^2+^ 3.21^d^

Galena [PbS] + H^+^ 🡘 Pb^2+^ + HS^-^ 14.85^d^

*Arsenic solids*

Realgar [AsS] + 2.5H_2_O + 0.25O_2_*_(aq)_* 🡘 H_2_AsO_3_^-^ + 2H^+^ + HS^-^ 7.80^g^

Orpiment [As_2_S_3_] + 6H_2_O 🡘 2H_2_AsO_3_^-^ + 3HS^-^ + 5H^+^ 64.6^g^

Arsenopyrite 14.44 ^g^

[FeAsS] +7H_2_O 🡘 H_3_AsO_3_^-^ + HS^-^ + Fe^2+^ +3H^+^ + 3e^-^

^a^ ([Delany and Lundeen, 1990](#_ENREF_4)); ^b^ ([Bigham et al., 1996](#_ENREF_2)); ^c^ alternate log *K*sp from ([Majzlan et al., 2004](#_ENREF_12)); ^d^ ([Johnson et al., 1991](#_ENREF_8)); ^e^ ([Wagman, 1982](#_ENREF_16)); ^f^ ([Kashkay et al., 1975](#_ENREF_9)); ^g^ ([Nordstrom and Archer, 2003](#_ENREF_13)).

**Table S3:** Selective Sequential Extraction Data for As, Pb, Zn, Mg, Al, K, Ti, Mn, and Cr.

|  | Element | Det. Limit | Total | DI H2O | NH4NO3 | AAc | PO4 | AAO | CBD | Σ(1-6) | Labile | Residual |
| --- | --- | --- | --- | --- | --- | --- | --- | --- | --- | --- | --- | --- |
|  |  | umol kg^-1^ | mmol kg^-1^ | umol kg^-1^ | umol kg^-1^ | umol kg^-1^ | umol kg^-1^ | umol kg^-1^ | umol kg^-1^ | umol kg^-1^ | % | % |
|  | As |  |  | Water Soluble | Plant Available | Acid Soluble | Specifically Sorbed | Amorphous oxy(hydr)oxides | Crystalline oxy(hydr)oxides | Extractable |  | Unextractable |
| Depth (cm) | A 0-5 | 51.9(3) | 41.1 | 37.5(9) | 2.22(2) | 107(160) | 6430(720) | 15200(1000) | 12600(600) | 34300(1400) | 83.5 | 16.5 |
|  | B 5-15 | 51.8(3) | 37.6 | 188(5) | 7.96(7) | 195(59) | 7390(710) | 27000(2000) | 11300(760) | 46100(2200) | 123. | -22.6 |
|  | C 15-25 | 51.5(2) | 54.4 | 24.2(6) | 5.11(4) | 176(14) | 4730(180) | 30200(1700) | 5480(920) | 40700(1900) | 74.8 | 25.2 |
|  | D 25-35 | 52.8(16) | 53.2 | 7.69(7) | 2.92(5) | 83.5(16) | 3470(250) | 19900(930) | 765(350) | 24300(1000) | 45.6 | 54.4 |
|  | E 35-38 | 50.0(29) | 70.1 | 1.40(1) | 8.94(3) | 189(22) | 3220(1600) | 9930(3300) | 137(57) | 13500(3700) | 19.2 | 80.8 |
|  | F 38-54 | 51.7(4) | 64.6 | 6.04(3) | 11.5(4) | 416(22) | 4950(360) | 7170(380) | 622(96) | 13200(540) | 20.4 | 79.6 |
|  | G 180-183 | 51.8(4) | 44.3 | <DL | 6.04(6) | 179(92) | 2280(420) | 4670(230) | 146(51) | 7280(490) | 16.4 | 83.6 |
|  |  |  |  |  |  |  |  |  |  |  |  |  |
|  | Element | Det. Limit | Total | DI H2O | NH4NO3 | AAc | PO4 | AAO | CBD | Σ(1-6) | Labile | Residual |
|  |  | umol kg^-1^ | mmol kg^-1^ | umol kg^-1^ | umol kg^-1^ | umol kg^-1^ | umol kg^-1^ | umol kg^-1^ | umol kg^-1^ | umol kg^-1^ | % | % |
|  | Pb |  |  | Water Soluble | Plant Available | Acid Soluble | Specifically Sorbed | Amorphous oxy(hydr)oxides | Crystalline oxy(hydr)oxides | Extractable |  | Unextractable |
| Depth (cm) | A 0-5 | 18.8(1) | 10.6 | 2.22(1) | 6.71(5) | 13.2(4) | 6.07(7) | 537(48) | 509(180) | 1070(190) | 10.1 | 89.9 |
|  | B 5-15 | 18.7(1) | 13.6 | 9.10(1) | 7.84(2) | 10.6(4) | 6.29(4) | 835(96) | 261(120) | 1130(160) | 8.3 | 91.7 |
|  | C 15-25 | 18.6(1) | 12.8 | 13.9(0.1) | 10.6(6) | 256(35) | 9.44(6) | 841(160) | 615(62) | 1750(170) | 13.6 | 86.4 |
|  |  | 19.1(6) | 8.06 | 2.72(0.3) | 5.34(4) | 222(7) | 8.18(5) | 1000(300) | 209(120) | 1450(330) | 18.0 | 82.0 |
|  | E 35-38 | 18.1(10) | 11.5 | 0.632(0.5) | 6.52(4) | 526(11) | 14.3(12) | 275(79) | 97.5(30) | 921(87) | 8.0 | 92.0 |
|  | F 38-54 | 18.7(1) | 12.7 | 11.0(1) | 11.3(8) | 2910(110) | 16.0(11) | 151(46) | 253(85) | 3350(150) | 26.4 | 73.6 |
|  | G 180-183 | 18.7(1) | 11.9 | 8.59(0.9) | 21.8(6) | 4190(180) | 23.4(15) | 62.1(9) | 158(26) | 4460(190) | 37.5 | 62.5 |

**Table S3 (con’t).**

|  | Element | Det. Limit | Total | DI H2O | NH4NO3 | AAc | | PO4 | | AAO | | CBD | | Σ(1-6) | Labile | | | Residual | | | |  |
| --- | --- | --- | --- | --- | --- | --- | --- | --- | --- | --- | --- | --- | --- | --- | --- | --- | --- | --- | --- | --- | --- | --- |
|  |  | umol kg^-1^ | mmol kg^-1^ | umol kg^-1^ | umol kg^-1^ | umol kg^-1^ | | umol kg^-1^ | | umol kg^-1^ | | umol kg^-1^ | | umol kg^-1^ | % | | | % | | | |  |
|  | Zn |  |  | Water Soluble | Plant Available | Acid Soluble | | Specifically Sorbed | | Amorphous oxy(hydr)oxides | | Crystalline oxy(hydr)oxides | | Extractable |  | | | Unextractable | | | |  |
| Depth (cm) | A 0-5 | 59.5(4) | 39.3 | 5100(11) | 343(160) | 150(30) | | 20.2(19) | | 5930(440) | | 15400(770) | | 26900(900) | 68.5 | | | 31.5 | | | |  |
|  | B 5-15 | 59.3(3) | 34.9 | 14000(270) | 869(260) | 181(62) | | 6.50(11) | | 9180(660) | | 17200(960) | | 41300(1200) | 118.5 | | | -18.5 | | | |  |
|  | C 15-25 | 59.1(3) | 79.2 | 23400(310) | 1480(310) | 3570(390) | | 3020(1700) | | 18800(1300) | | 5200(1200) | | 55400(2500) | 70.0 | | | 30.0 | | | |  |
|  | D 25-35 | 60.4(19) | 86.2 | 24600(180) | 2130(790) | 12300(600) | | 3090(400) | | 21900(1200) | | 786(96) | | 64800(1600) | 75.2 | | | 24.8 | | | |  |
|  | E 35-38 | 57.3(33) | 168 | 14800(800) | 3910(290) | 26100(1000) | | 6000(2900) | | 20300(6400) | | 663(94) | | 71800(7200) | 42.7 | | | 57.3 | | | |  |
|  | F 38-54 | 59.2(4) | 148 | 54800(160) | 8210(500) | 53700(1200) | | 7270(1900) | | 25200(2600) | | 1440(800) | | 150000(3600) | 101.8 | | | -1.8 | | | |  |
|  | G 180-183 | 59.4(4) | 107 | 5450(220) | 2190(190) | 34600(9700) | | 12300(2200) | | 32900(2400) | | 541(26) | | 88000(10000) | 82.3 | | | 17.7 | | | |  |
|  |  |  |  |  |  |  | | |  | |  | |  |  | | |  | | |  | | |
|  | Element | Det. Limit | Total | DI H2O | NH4NO3 | AAc | | PO4 | | AAO | | CBD | | Σ(1-6) | Labile | | | | Residual | |  |  |
|  |  | umol kg^-1^ | mmol kg^-1^ | umol kg^-1^ | umol kg^-1^ | umol kg^-1^ | | umol kg^-1^ | | umol kg^-1^ | | umol kg^-1^ | | umol kg^-1^ | % | | | | % | |  |  |
|  | Mg |  |  | Water Soluble | Plant Available | Acid Soluble | | Specifically Sorbed | | Amorphous oxy(hydr)oxides | | Crystalline oxy(hydr)oxides | | Extractable |  | | | | Unextractable | |  |  |
| Depth (cm) | A 0-5 | 160(10) | 293 | 12400(100) | 41700(52000) | | 22.5(24) | | 816(94) | | 468(110) | | 960(880) | 56400(52000) | | 19.3 | | | 80.7 | |  |  |
|  | B 5-15 | 160(8) | 320 | 26800(770) | 60300(29000) | | 102(69) | | 1660(700) | | 1570(670) | | 798(240) | 91200(29000) | | 28.5 | | | 71.5 | |  |  |
|  | C 15-25 | 159(7) | 350 | 30500(570) | 226000(17000) | | 6780(210) | | 9850(7100) | | 9900(550) | | 2920(800) | 286000(18000) | | 81.6 | | | 18.4 | |  |  |
|  | D 25-35 | 163(50) | 320 | 25300(390) | 365000(65000) | | 35700(4400) | | 5500(1800) | | 41800(1500) | | 5090(370) | 478000(66000) | | 149.5 | | | -49.5 | |  |  |
|  | E 35-38 | 154(89) | 791 | 40300(1800) | 249000(24000) | | 68800(5600) | | 10300(5400) | | 72900(21000) | | 7500(770) | 449000(33000) | | 56.8 | | | 43.2 | |  |  |
|  | F 38-54 | 159(11) | 854 | 116000(930) | 391000(54000) | | 68800(4300) | | 13000(1200) | | 110000(5200) | | 11000(3400) | 710000(55000) | | 83.2 | | | 16.8 | |  |  |
|  | G 180-183 | 160(12) | 633 | 101000(2300) | 200000(45000) | | 87500(37000) | | 13300(2400) | | 82600(2300) | | 5930(600) | 491000(58000) | | 77.5 | | | 22.5 | |  |  |

**Table S3 (con’t)**

|  | Element | Det. Limit | Total | DI H_2_O | NH_4_NO_3_ | AAc | NaH_2_PO_4_ | AAO | CBD | Σ(1-6) | Labile | Residual |
| --- | --- | --- | --- | --- | --- | --- | --- | --- | --- | --- | --- | --- |
|  |  | umol kg^-1^ | mmol kg^-1^ | umol kg^-1^ | umol kg^-1^ | umol kg^-1^ | umol kg^-1^ | umol kg^-1^ | umol kg^-1^ | umol kg^-1^ | % | % |
|  | Al |  |  | Water Soluble | Plant Available | Acid Soluble | Specifically Sorbed | Amorphous oxy(hydr)oxides | Crystalline oxy(hydr)oxides | Extractable |  | Unextractable |
| Depth | A 0-5 | 144(9) | 788 | 17300(470) | 7960(13000) | 509(250) | 356(250) | 4560(880) | 9240(340) | 39900(13000) | 5.1 | 94.9 |
|  | B 5-15 | 144(7) | 824 | 44800(1500) | 1400(870) | 865(280) | 1450(440) | 10800(760) | 13100(1400) | 72400(2400) | 8.8 | 91.2 |
|  | C 15-25 | 143(6) | 822 | 49400(1400) | 390(420) | 31200(2400) | 12800(1200) | 125000(9200) | 14400(5800) | 233000(11000) | 28.4 | 71.6 |
|  | D 25-35 | 146(45) | 1060 | 4960(280) | 1790(3000) | 14900(1500) | 13800(2200) | 83100(1100) | 2730(890) | 121000(4200) | 11.4 | 88.6 |
|  | E 35-38 | 139(80) | 1620 | 150(260) | 275(72) | 1700(480) | 3570(1900) | 5150(1600) | 980(340) | 11800(2600) | 0.7 | 99.3 |
|  | F 38-54 | 144(10) | 1100 | 2350(4100) | 363(400) | 1060(330) | 6520(3700) | 11200(1000) | 1560(530) | 23000(5600) | 2.1 | 97.9 |
|  | G 180-183 | 144(11) | 378 | 185(320) | 349(300) | 7190(9600) | 9370(6300) | 11900(800) | 3220(930) | 32200(12000) | 8.5 | 91.5 |
|  |  |  |  |  |  |  |  |  |  |  |  |  |
|  | Element | Det. Limit | Total | DI H_2_O | NH_4_NO_3_ | AAc | NaH_2_PO_4_ | AAO | CBD | Σ(1-6) | Labile | Residual |
|  |  | umol kg^-1^ | mmol kg^-1^ | umol kg^-1^ | umol kg^-1^ | umol kg^-1^ | umol kg^-1^ | umol kg^-1^ | umol kg^-1^ | umol kg^-1^ | % | % |
|  | K |  |  | Water Soluble | Plant Available | Acid Soluble | Specifically Sorbed | Amorphous oxy(hydr)oxides | Crystalline oxy(hydr)oxides | Extractable |  | Unextractable |
| Depth | A 0-5 | 99.5(6) | 84.9 | <DL | 28400(33000) | 1200(1200) | 772(1300) | 1340(2300) | 12400(7400) | 44100(34000) | 51.9 | 48.1 |
|  | B 5-15 | 99.2(5) | 70.1 | 95.3(160) | 20600(32000) | 458(790) | 406(700) | 1060(940) | 764(640) | 23400(33000) | 33.3 | 66.7 |
|  | C 15-25 | 98.8(4) | 76.4 | 155(270) | 29200(50000) | 971(860) | 3180(4800) | 117(200) | 2670(2900) | 36300(51000) | 47.5 | 52.5 |
|  | D 25-35 | 101(31) | 59.4 | 153(260) | 5450(9400) | 385(470) | 3340(2800) | 39.5(68) | 5070(7200) | 14400(12000) | 24.3 | 75.7 |
|  | E 35-38 | 95.8(55) | 136 | 206(220) | 23100(33000) | 276(340) | 2950(5100) | 48.5(84) | 2050(1900) | 28600(34000) | 21.1 | 78.9 |
|  | F 38-54 | 99.0(7) | 115 | 980(1700) | 23600(35000) | 1020(880) | 3140(4200) | 413(710) | 2760(3000) | 31900(35000) | 27.7 | 72.3 |
|  | G 180-183 | 99.3(7) | 61.6 | 199(340) | 5650(9200) | 4800(5900) | 3930(4200) | 986(1700) | 2190(3000) | 17800(12000) | 28.8 | 71.2 |

**Table S3 (con’t)**

|  | Element | Det. Limit | Total | DI H_2_O | NH_4_NO_3_ | AAc | NaH_2_PO_4_ | AAO | CBD | Σ(1-6) | Labile | Residual |
| --- | --- | --- | --- | --- | --- | --- | --- | --- | --- | --- | --- | --- |
|  |  | umol kg^-1^ | mmol kg^-1^ | umol kg^-1^ | umol kg^-1^ | umol kg^-1^ | umol kg^-1^ | umol kg^-1^ | umol kg^-1^ | umol kg^-1^ | % | % |
|  | Ti |  |  | Water Soluble | Plant Available | Acid Soluble | Specifically Sorbed | Amorphous oxy(hydr)oxides | Crystalline oxy(hydr)oxides | Extractable |  | Unextractable |
| Depth | A 0-5 | 81.3(5) | 68.6 | <DL | 4990(7200) | <DL | <DL | 386(60) | 324(62) | 5760(7200) | 8.4 | 91.6 |
|  | B 5-15 | 81.0(4) | 89.9 | <DL | 2650(3100) | <DL | <DL | 345(41) | 180(59) | 3240(3100) | 3.6 | 96.4 |
|  | C 15-25 | 80.7(4) | 68.4 | <DL | 2490(1900) | <DL | <DL | 154(39) | <DL | 2790(1900) | 4.1 | 95.9 |
|  | D 25-35 | 82.6(25) | 59.8 | <DL | 1330(220) | <DL | <DL | 121(30) | <DL | 1500(220) | 2.5 | 97.5 |
|  | E 35-38 | 78.2(45) | 59.7 | <DL | 1200(400) | <DL | <DL | 96.0(56) | 90.3(35) | 1440(410) | 2.4 | 97.6 |
|  | F 38-54 | 80.9(6) | 64.0 | <DL | 1450(890) | <DL | <DL | 85.7(38) | <DL | 1660(890) | 2.6 | 97.4 |
|  | G 180-183 | 81.1(6) | 42.2 | <DL | 518(460) | <DL | <DL | 108(73) | 61.7(41) | 727(470) | 1.7 | 98.3 |
|  |  |  |  |  |  |  |  |  |  |  |  |  |
|  | Element | Det. Limit | Total | DI H_2_O | NH_4_NO_3_ | AAc | NaH_2_PO_4_ | AAO | CBD | Σ(1-6) | Labile | Residual |
|  |  | umol kg^-1^ | mmol kg^-1^ | umol kg^-1^ | umol kg^-1^ | umol kg^-1^ | umol kg^-1^ | umol kg^-1^ | umol kg^-1^ | umol kg^-1^ | % | % |
|  | Mn |  |  | Water Soluble | Plant Available | Acid Soluble | Specifically Sorbed | Amorphous oxy(hydr)oxides | Crystalline oxy(hydr)oxides | Extractable |  | Unextractable |
| Depth | A 0-5 | 70.8(4) | 3.81 | 358(7) | 87.2(24) | <DL | <DL | 851(60) | 3370(86) | 4690(110) | 123 | -23.1 |
|  | B 5-15 | 70.6(4) | 6.34 | 650(26) | 174(16) | <DL | <DL | 300(18) | 2000(150) | 3150(150) | 49.7 | 50.3 |
|  | C 15-25 | 70.3(3) | 6.34 | 1420(15) | 243(15) | 426(46) | 232(240) | 1190(110) | 203(58) | 3710(270) | 58.6 | 41.4 |
|  | D 25-35 | 71.9(22) | 3.81 | 2250(40) | 537(143) | 1630(99) | 280(49) | 2450(76) | 404(32) | 7550(200) | 198 | -98.3 |
|  | E 35-38 | 68.2(40) | 20.2 | 3840(210) | 856(50) | 3990(210) | 914(440) | 4380(1400) | 1100(110) | 15100(1500) | 74.7 | 25.3 |
|  | F 38-54 | 70.4(5) | 29.9 | 12700(100) | 2490(170) | 8860(160) | 847(220) | 6360(670) | 1720(590) | 33000(950) | 110 | -10.4 |
|  | G 180-183 | 70.7(5) | 11.8 | 3000(64) | 1160(54) | 6440(1500) | 1570(260) | 6770(440) | 1310(250) | 20200(1600) | 172 | -71.6 |

**Table S3 (con’t)**

|  | Element | Det. Limit | Total | DI H_2_O | NH_4_NO_3_ | AAc | NaH_2_PO_4_ | AAO | CBD | Σ(1-6) | Labile | Residual |
| --- | --- | --- | --- | --- | --- | --- | --- | --- | --- | --- | --- | --- |
|  |  | umol kg^-1^ | mmol kg^-1^ | umol kg^-1^ | umol kg^-1^ | umol kg^-1^ | umol kg^-1^ | umol kg^-1^ | umol kg^-1^ | umol kg^-1^ | % | % |
|  | Cr |  |  | Water Soluble | Plant Available | Acid Soluble | Specifically Sorbed | Amorphous oxy(hydr)oxides | Crystalline oxy(hydr)oxides | Extractable |  | Unextractable |
| Depth | A 0-5 | 74.8(5) | 0.6 | <DL | <DL | <DL | <DL | <DL | 476(810) | 530(810) | 83.5 | 16.5 |
|  | B 5-15 | 74.6(4) | 0.7 | <DL | <DL | <DL | <DL | 167(29) | 197(300) | 457(310) | 61.2 | 35.8 |
|  | C 15-25 | 74.3(3) | 0.5 | <DL | <DL | <DL | <DL | 163(12) | <DL | 215(27) | 42.9 | 57.1 |
|  | D 25-35 | 76.0(23) | 0.7 | <DL | <DL | <DL | <DL | 116(17) | 463(780) | 617(780) | 84.4 | 15.6 |
|  | E 35-38 | 72.0(42) | 1.2 | <DL | <DL | <DL | <DL | 84.9(48) | <DL | 113(51) | 9.45 | 90.6 |
|  | F 38-54 | 74.4(5) | 0.7 | <DL | <DL | <DL | <DL | 167(20) | 1380(2300) | 1590(2300) | 230 | -130 |
|  | G 180-183 | 74.7(6) | 0.6 | <DL | <DL | <DL | <DL | 131(5) | 241(330) | 462(330) | 80.8 | 19.2 |

**Figure S1: Multiple Energy XANES Mapping Energies**

**
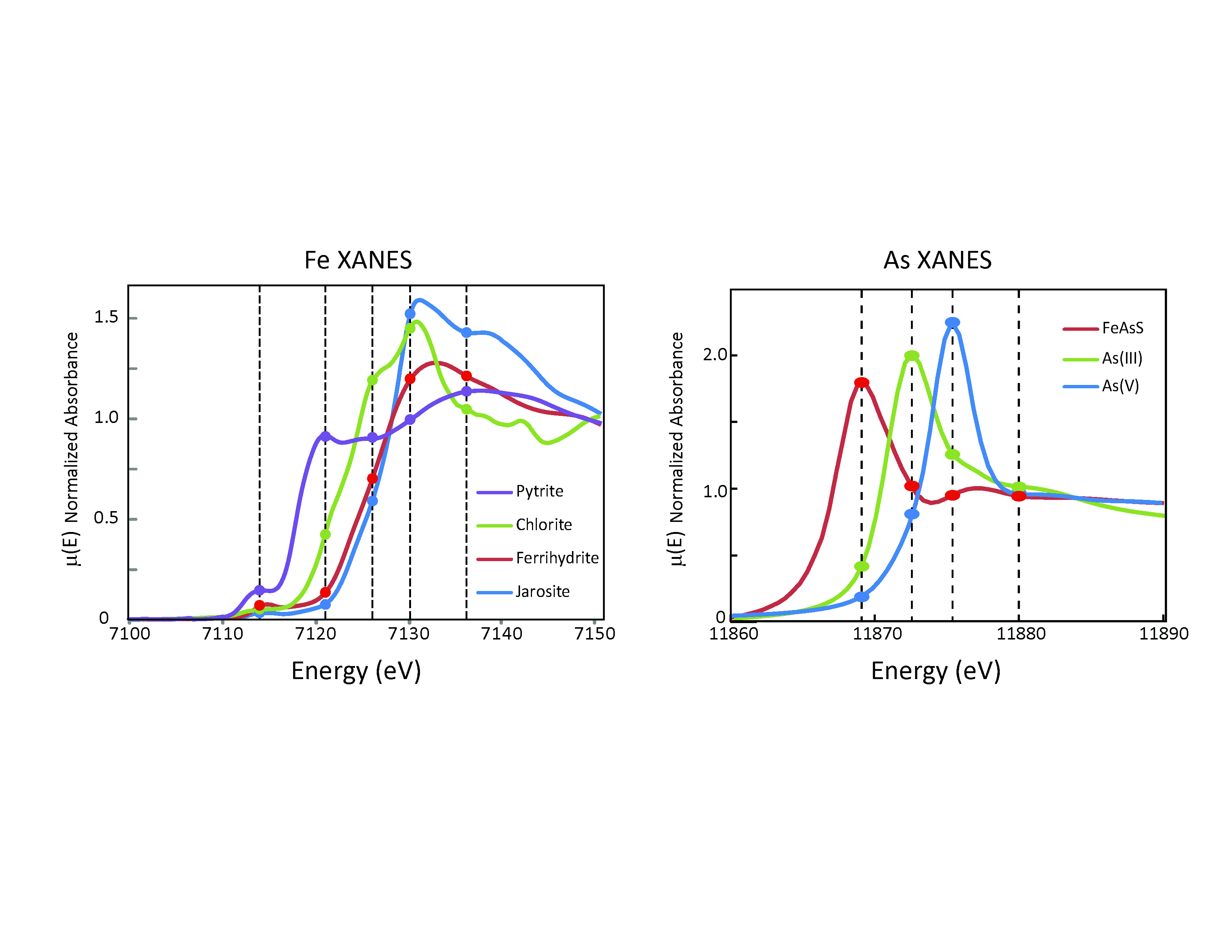
Figure S1:** Calibrated energy (eV) for multiple energy micro X-ray fluorescence (ME-μXRF) imaging, aka XANES mapping, Fe and As. Dashed lines indicate the energy of individual maps, line symbols indicate the fluorescence response of model minerals, shown in solid lines (see online version for color reference) that are entered into an energy v. fluorescence matrix where F*_m_* = Σc_i,j_ ω_i,j_. Fluorescence measured (F*m*) is the sum of the concentration of each chemical (c_i_) or species (c_j_) time the fluorescence response of the element (ω_i_)or species (ω_j_) at the specific energy used to probe the sample. Combining the maps results in each pixel having five Fe energies or four As energies to build a XANES map that is sensitive to oxidation state and mineral species.

**Figure S2: Elemental correlations and p-values**

**
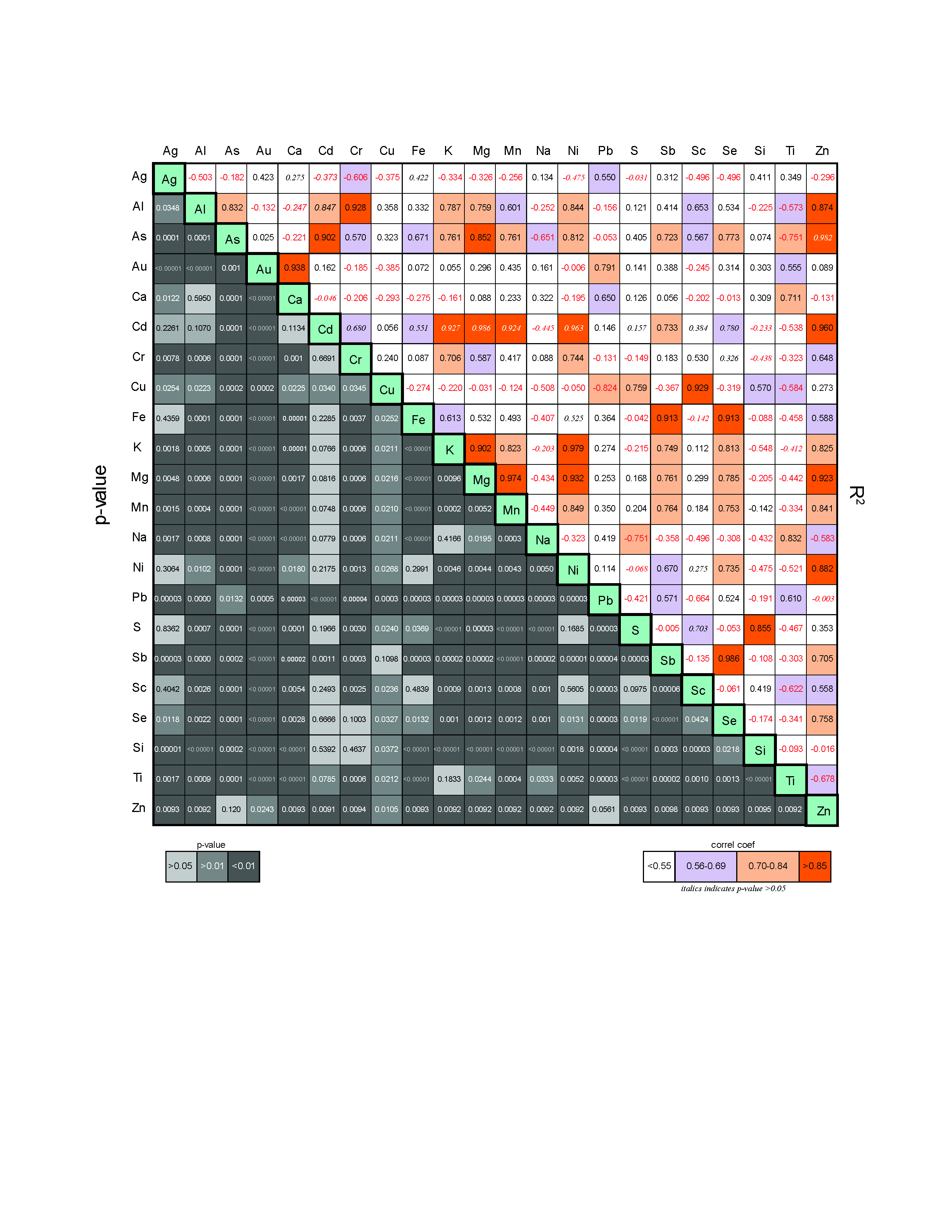
**

**Figure S2:** Total mass concentrations for a suite of elements was measured by fusion ICP-MS for the IKMHSS tailings from 0-1m at four coring locations across a 10,000 m^2^ on top of the tailings pile (n=16) within a phytostabilization trial (at t=0). Correlations are between elements at subsectioned depths: 0-20 cm; 20-40 cm; 40-60 cm; 60-100 cm. are shown on the upper right portion of the plot, and p-values for the corresponding correlations are shown on the lower left of the plot. Color intensity directs the reader to the highest correlations and lowest p-values.

**Figure S3: As XANES during SSE**

**
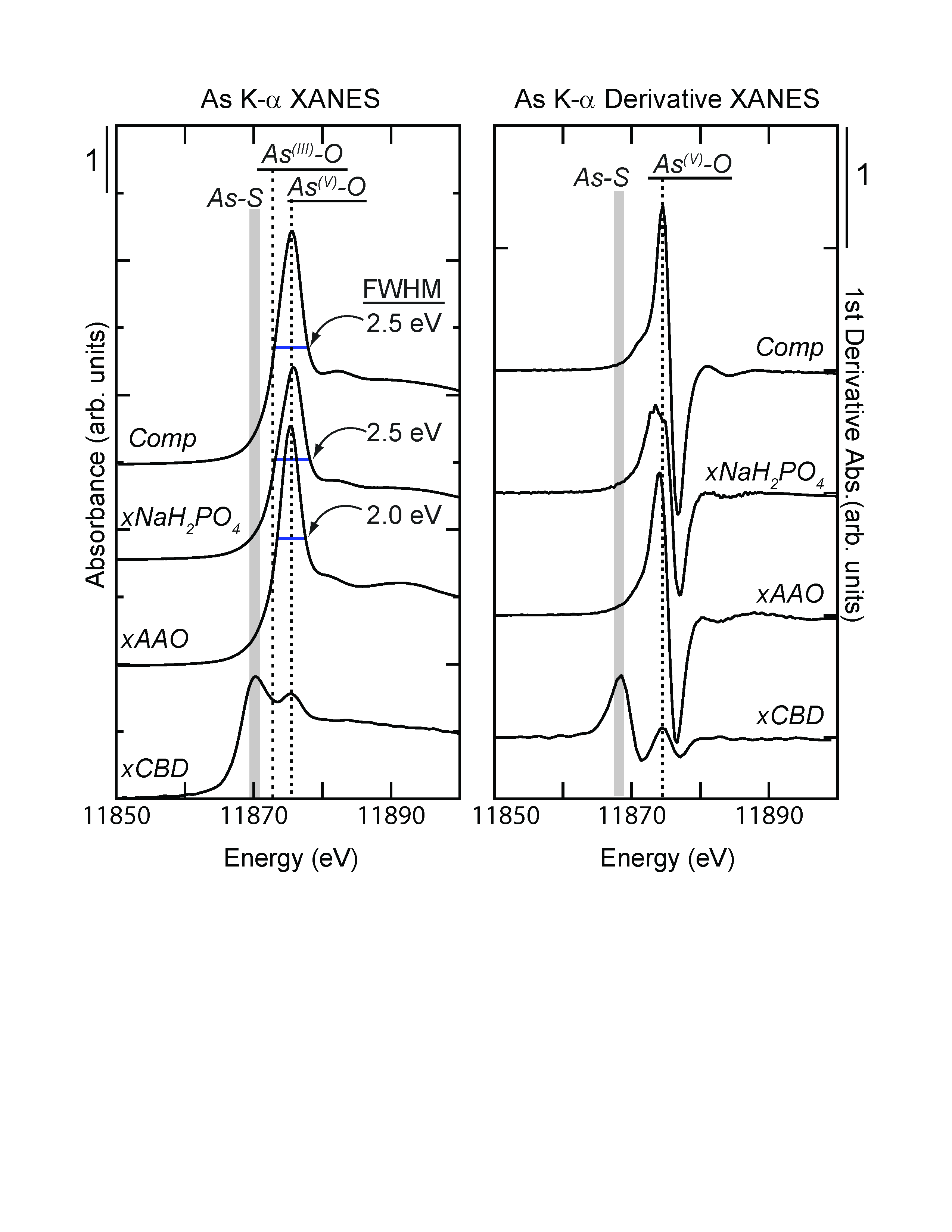
**

**Figure S5:** Difference spectra for As K-edge XANES are shown for the 0-25 cm composite IKMHSS sample before and during sequential extraction. The peak shape and sharpness, defined by the full width half the maximum intensity (FWHM) is unchanged after the NaH_2_PO_4_ extraction, targeting surface complex As(V) in Fe octahedra, but reduces by 20% after the ammonium oxalate step. The broadening of the main peak (white-line) can be attributed artifacts of differences in self absorption (concentration), sample thickness, or inherent beamline differences. The spectra were collected at the same beamline during the same beam run, which discounts hardware differences. Self absorption and sample thickness are rule out by concurrent examination of the transmission signal. The peak sharpening is attributed to the dissolution of poorly ordered As sorbed ferrihydrite and a relative concentration in As associated with a more crystalline phase. The post CBD spectrum shows mostly arsenopyrite remains, but there is a contribution of As(V) that is recalcitrant after the extraction targeting reducible iron. The removal of ferrihydrite associated As is inferred from the AAO step, the removal of jarosite associated As is inferred from the CBD step and the residual As(V) after CBD is likely sequestered as sulfate substitution in jarosite.

**References**

Anderson, C.A., Creasey, S.C., 1958. Geology and ore deposites of teh Jerome area, Yavapai County, Arizona. In: USGS (Editor), Prof. Paer 308, pp. 185.

Bigham, J.M., Schwertmann, U., Traina, S.J., Winland, R.L., Wolf, M., 1996. Schwertmannite and the chemical modeling of iron in acid sulfate waters. Geochimica et Cosmochimica Acta, 60: 2111-2121.

Creasey, S.C., 1952. Geology of Iron King mine, Yavapai County, Arizona. Economic Geology, 47(1): 24-56.

Delany, J.M., Lundeen, S.R., 1990. The LLNL Thermochemical Database, Reportt UCRL-21658. Lawrence Livermore National Laboratory

Hayes, S.M., O'Day, P.A., Webb, S.M., Maier, R.M., Chorover, J., 2011. Spectroscopic investigation of zinc speciation changes with pH in arid mine tailings in Aravaipa Canyon, Arizona. Environmental Science & Technology, 45: 7168-7172.

Hayes, S.M. et al., 2012. Geochemical Weathering Increases Lead Bioaccessibility in Semi-Arid Mine Tailings. Environmental Science & Technology, 46(11): 5834-5841.

Hayes, S.M., White, S.A., Thompson, T.L., Maier, R.M., Chorover, J., 2009. Changes in lead and zinc lability during weathering-induced acidification of desert mine tailings: Coupling chemical and micro-scale analyses. Applied Geochemistry, 24(12): 2234-2245.

Johnson, J.W., Oelkers, E.H., Helgeson, H.C., 1991. SUPCRT92: A software package for calculating the standard molal thermodynamic properties of minerals, gases, aqueous species, and reactions from 1 to 5000 bars and 0° to 1000°C. Earth Sciences Department, Lawrence Livermore Laboratory, pp. 101.

Kashkay, C.M., Borovskaya, Y.B., Babazade, M.A., 1975. Determination of deltaGf 298 of synthetic jarosite and its sulfate analogues. Geokhimiya, 5: 778-784.

Koschmann, A., Bergendahl, M., 1968a. Principal gold-producing distrcits of the United States. Professional Paper 610. USGS.

Koschmann, A.H., Bergendahl, M.H., 1968b. Principal gold-producing distrcits of the United States. In: USGS (Editor), Prof. Paer 610, pp. 283.

Majzlan, J., Navrotsky, A., Schwertmann, U., 2004. Thermodynamics of iron oxides. III - Enthalpies of formation oand stability of ferrihydrite (Fe(OH)3), schwertmannite (FeO(OH)3/4(SO4)i/8), and Fe2O3. Geochimica et Cosmochimica Acta, 68: 1049-1059.

Nordstrom, D.K., Archer, D.G., 2003. *Arsenic thermodynamic data and environmental geochemistry*. In: Welch, A.H., Stollenwerk, K.G. (Eds.). Kluwer Academic Publishers, Dordrecht, pp. 1-25.

O'Day, P.A., Rivera, N., Root, R., Carroll, S.A., 2004. X-ray absorption spectroscopic study of Fe reference compounds for the analysis of natural sediments. American Mineralogist, 89(4): 572-585.

USEPA, 2014. Iron King Mine and Humboldt Smelter. In: 9, R. (Editor). USEPA.

Wagman, D.D., Evans, W.H., Parker, V.B., Schumm, R.H., Halow, I., 1982. The NBS Tables of Chemical Thermodynamic Properties. Selected Values for Inorganic and C1 and C2 Organic Substances in SI Units. National Standard Reference Data System, Gaithersburg, MD.

1. [↑](#footnote-ref-1)
